# Cerebrospinal Fluid Biomarkers of Alzheimer’s Disease: Current Evidence and Future Perspectives

**DOI:** 10.3390/brainsci11020215

**Published:** 2021-02-10

**Authors:** Donovan A. McGrowder, Fabian Miller, Kurt Vaz, Chukwuemeka Nwokocha, Cameil Wilson-Clarke, Melisa Anderson-Cross, Jabari Brown, Lennox Anderson-Jackson, Lowen Williams, Lyndon Latore, Rory Thompson, Ruby Alexander-Lindo

**Affiliations:** 1 Department of Pathology, Faculty of Medical Sciences, The University of the West Indies, Kingston 7, Jamaica; kurt.vaz@uwimona.edu.jm (K.V.); jabarigbrown@gmail.com (J.B.); lennoxwaj@hotmail.com (L.A.-J.); l_latore@yahoo.com (L.L.); rorykthompson@gmail.com (R.T.); 2 Department of Physical Education, Faculty of Education, The Mico University College, 1A Marescaux Road, Kingston 5, Jamaica; miller9fabian_gov@yahoo.com; 3 Department of Biotechnology, Faculty of Science and Technology, The University of the West Indies, Kingston 7, Jamaica; williamslowen@yahoo.com; 4 Department of Basic Medical Sciences, Faculty of Medical Sciences, The University of the West Indies, Kingston 7, Jamaica; chukwuemeka.nwokocha@uwimona.edu.jm (C.N.); cameil.wilsonclarke@uwimona.edu.jm (C.W.-C.); lisa.lindo@uwimona.edu.jm (R.A.-L.); 5 School of Allied Health and Wellness, College of Health Sciences, University of Technology, Kingston 7, Jamaica; melisa_anderson4life@yahoo.com

**Keywords:** Alzheimer’s disease, biomarkers, amyloid, tau, cerebrospinal fluid

## Abstract

Alzheimer’s disease is a progressive, clinically heterogeneous, and particularly complex neurodegenerative disease characterized by a decline in cognition. Over the last two decades, there has been significant growth in the investigation of cerebrospinal fluid (CSF) biomarkers for Alzheimer’s disease. This review presents current evidence from many clinical neurochemical studies, with findings that attest to the efficacy of existing core CSF biomarkers such as total tau, phosphorylated tau, and amyloid-β (Aβ_42_), which diagnose Alzheimer’s disease in the early and dementia stages of the disorder. The heterogeneity of the pathophysiology of the late-onset disease warrants the growth of the Alzheimer’s disease CSF biomarker toolbox; more biomarkers showing other aspects of the disease mechanism are needed. This review focuses on new biomarkers that track Alzheimer’s disease pathology, such as those that assess neuronal injury (VILIP-1 and neurofilament light), neuroinflammation (sTREM2, YKL-40, osteopontin, GFAP, progranulin, and MCP-1), synaptic dysfunction (SNAP-25 and GAP-43), vascular dysregulation (hFABP), as well as CSF α-synuclein levels and TDP-43 pathology. Some of these biomarkers are promising candidates as they are specific and predict future rates of cognitive decline. Findings from the combinations of subclasses of new Alzheimer’s disease biomarkers that improve their diagnostic efficacy in detecting associated pathological changes are also presented.

## 1. Introduction

### Alzheimer’s Disease

Dementia defines a collection of symptoms due to acquired loss of cognition in numerous domains in such a manner that affects memory, occupational function, and thinking ability. The impairment in cognition significantly interferes with an individual’s ability to maintain normal daily activity and live a fully autonomous and purposeful life [1]. Globally, Alzheimer’s disease is the most common cause of dementia, encompassing 60–80% of cases. It has an estimated prevalence of over 40 million individuals worldwide and is reported to be the sixth leading cause of death [2]. The incidence of Alzheimer’s disease escalates with age, especially in an aging population, and the average duration of the disease from diagnosis to death is approximately 10 years. In the United States of America, an estimated prevalence of 3% has been reported for persons 65–74 years of age, 17% for 75–84 years of age, and 32% for those individuals 85 years and older [3].

Alzheimer’s disease is a progressive, clinically heterogeneous, and particularly complex neurodegenerative disease characterized by a decline in cognition. The disease usually starts with episodic memory impairment, followed by a continuing loss of the instrumental and simple activities of day-to-day living [4]. Individuals display behavioral disturbances, and there are noncognitive neurological deficits. The pathological progression of Alzheimer’s disease perhaps begins decades prior to the onset of signs, symptoms, and clinical diagnosis [5]. The pathological characteristics of this disease comprise the buildup of beta-amyloid (Aβ) plaques, intracellular neurofibrillary tangles consisting of hyper-phosphorylated tau fibrils, degeneration of neurons and loss of synapses, neuroinflammation, and glial activation [6].

The Diagnostic and Statistical Manual of Mental Disorders (DSM-V) from the American Psychiatric Association and the International Classification of Diseases (ICD 10) of the World Health Organization clearly state the criteria for the diagnosis of Alzheimer’s disease [7]. Furthermore, using the inclusion and exclusion criteria, Alzheimer’s Disease and Related Disorders Association (ADRDA) guidelines, National Institute of Neurological and Communicative Disorders and Stroke (NINCDS) guidelines, ICD-10, and DSM-V can be used to diagnose Alzheimer’s disease when reliable biomarkers are unavailable [8]. ADRDA and NINCDS criteria establish the diagnosis of Alzheimer’s disease as definite, probable, or possible. However, Alzheimer’s disease can be reliably diagnosed with 90% certainty based on clinical criteria such as neuropsychological evaluation, neuroimaging laboratory tests (including blood and cerebrospinal fluid (CSF) biomarkers), and the clinical history of the individual [9].

Biomarkers are naturally occurring signatures of molecules, molecular markers, or biochemical transformations observed in biological media, such as fluids, cells, or human tissues, that give an indication of normal physiological state, pathological processes, or pharmacologic responses to therapy [10]. Biomarkers are isolated from serum, urine, or other body fluids such as CSF and are valuable for establishing the diagnosis of a disease and predicting the outcome; they also play an important role in the development of novel drug therapies [11].

An important objective of biomedical research related to Alzheimer’s disease is to identify sensitive and specific biological indicators (i.e., biomarkers) that facilitate early diagnosis and intervention [12]. These biomarkers should be able to be quantified with a reliable and standardized assay and accurately reflect the biological processes of the disease that are connected to clinical endpoints [13]. Over the last decade or so, there have been significant advances made in developing biomarkers that permit in-vivo evaluation of Alzheimer’s disease [14,15].

Alzheimer’s disease is categorized by neuroinflammation, mitochondrial impairment, synaptic dysfunction, oxidative stress, and disruption in the blood–brain barrier, which may be due to uncharacteristic extracellular buildup and deposition of the amyloid-β peptide (Aβ) in amyloid plaques and accumulation of neuronal hyperphosphorylated tau protein in intracellular neurofibrillary tangles (NFTs) [16]. This results in synaptic loss, neuronal loss (notably, the temporoparietal association cortices and medial temporal lobe structures), memory impairment, and enhanced cognitive dysfunction due to progressive neuronal degeneration [17,18].

There are two types of Alzheimer’s disease, classified according to pathological factors and the age of commencement. Early-onset or familial Alzheimer’s disease is multifactorial and has a sporadic occurrence. These patients usually present with other comorbidities such as hypertension, diabetes mellitus (type 1and 2), obesity, hypercholesterolemia, and metabolic syndrome [2]. In the second type—late-onset Alzheimer’s disease—there is an elevation of Aβ_42_ fragment levels, resulting in the formation of amyloid plaques and excitotoxicity. The pathological process of late-onset Alzheimer’s disease involves mitochondrial damage of neurons, oxidative stress, and apolipoprotein E (APOE) polymorphism in the vascular endothelium [19].

Existing biomarkers for the diagnosis of Alzheimer’s disease include (1) Aβ_42_ (the 42 amino acid form of amyloid β) in CSF and (2) total and phosphorylated tau protein in CSF [20]. The utilization of these biomarkers, with acceptable sensitivity and specificity, contribute to the early detection of Alzheimer’s disease, particularly in individuals with mild cognitive impairment, with higher diagnostic certainty [21].

The Amyloid/Tau/Neurodegeneration (A/T/N) system, a biomarker-based biological classification, was published in 2018 by a work group commissioned by the National Institute on Aging and the Alzheimer’s Association [22]. There is a reference to A (representing amyloid plaques) and T (tau neurofibrillary tangles); these biomarkers are precise neuropathological indicators of Alzheimer’s disease. The β-amyloid biomarker is assessed by measuring CSF Aβ_42_ levels using immunoassays for liquid chromatography–tandem mass spectrometry or detection of amyloid PET [23]. Phosphorylated tau has been measured extensively using enzyme-linked immunosorbent assays (ELISAs) or detected on tau positron emission tomography (PET) [24]. N represents biomarkers of neuronal injury or neurodegeneration, which are assessed by measuring total tau in CSF by ELISA or atrophy by magnetic resonance imaging (MRI) or [18F]-fluorodeoxyglucose PET; the latter not regarded as disease-specific [25]. There are respective cut-off points for A, T, and N, which are classified as normal (−) or abnormal (+), with, subsequently, eight different AT(N) biomarker profiles [26].

Over the last two decades, there has been significant growth in the investigation of cerebrospinal fluid (CSF) biomarkers for Alzheimer’s disease. This review presents current evidence from many clinical neurochemical studies on core and novel CSF biomarkers that assess all aspects of the pathophysiology of the disease.

## 2. Materials and Methods

**Figure 1:**
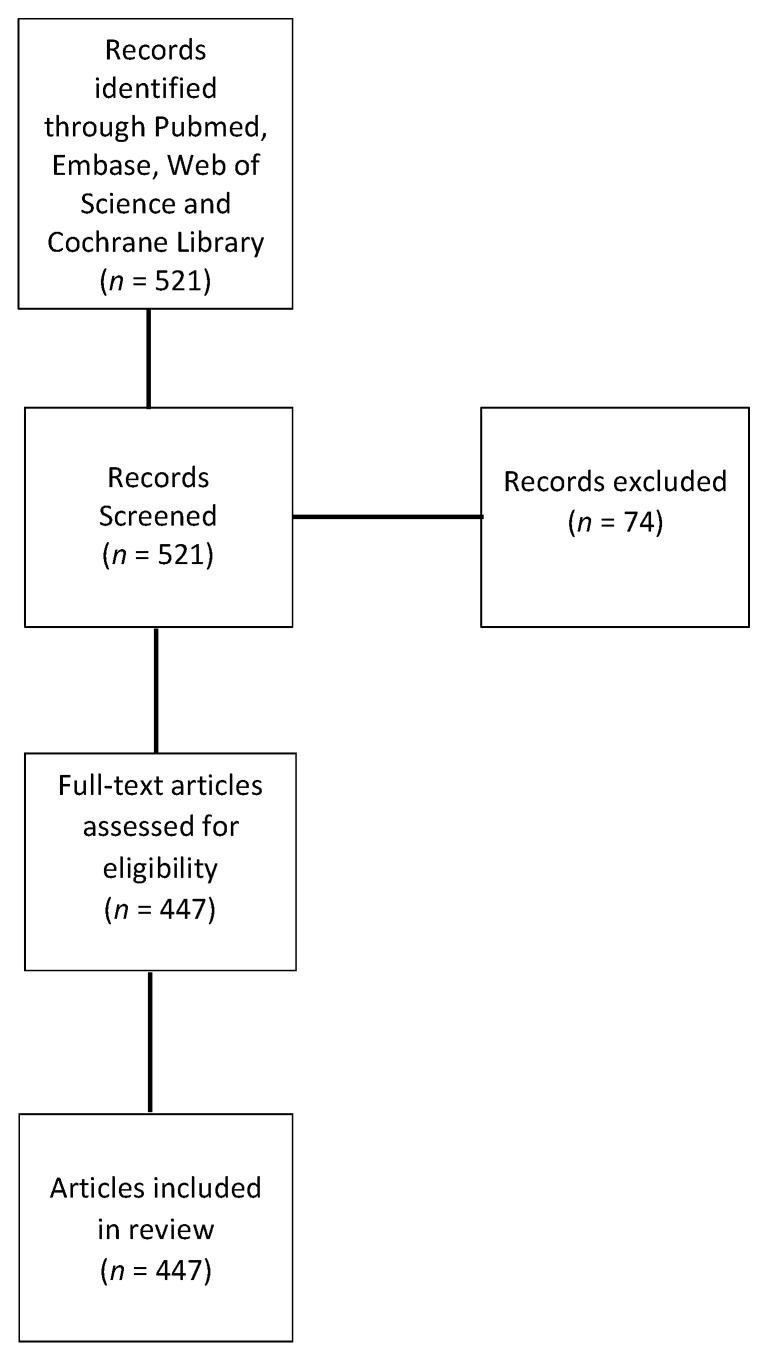
Flow chart of literature search and study selection.

A literature search was conducted for all English language literature published before December 2020. The search was conducted using electronic databases, including PubMed, Embase, Web of Science, and Cochrane Library. The search strategy included keywords such as Alzheimer’s disease, cerebrospinal fluid, biomarkers, beta-amyloid, tau, neurofilament light, neuroinflammation osteopontin, progranulin, synaptic dysfunction, vascular dysregulation α-synuclein levels, and TDP-43 pathology, amongst other search terms. Publications that were not in English were excluded due to the challenges of evaluating the contents as accessible open resources. The most relevant or primary studies were included in this analysis, and replicated contents were omitted along with studies with a small sample size (Figure 1). Studies with important findings of key CSF biomarkers are included in Table 1, Table 2 and Table 3. Of note, key findings of meta-analysis articles are given in Table 1.

## 3. Cerebrospinal Fluid Biomarkers of β-amyloid Aggregation, Metabolism, and Pathology in Alzheimer’s Disease

### 3.1. Cerebrospinal Fluid Aβ Peptides

**Figure 2:**
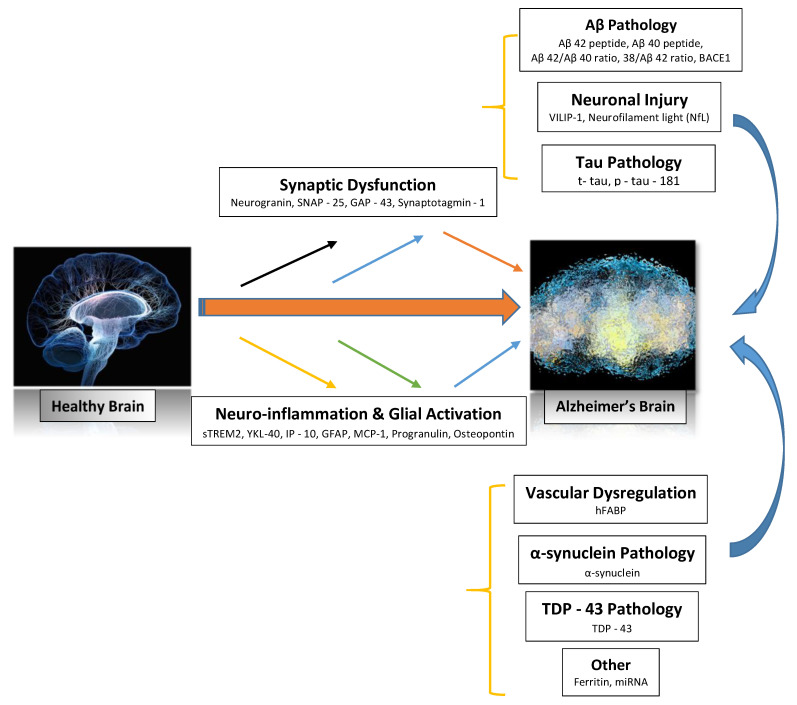
Pathological events and their cerebrospinal fluid biomarkers in Alzheimer’s disease.

Alzheimer’s disease is the most dominant neurodegenerative disorder, and the hallmark of its primary pathology involves the metabolism and extracellular deposition of β-amyloid (Aβ) peptides (Figure 2) [37]. The amyloid cascade hypothesis gives credence to the pathogenesis of Alzheimer’s disease, with the initial mechanistic event being the abnormal aggregation of β-amyloid (Aβ) peptides where soluble oligomers are transformed into insoluble fibers or plaques [38]. The progressive Aβ plaque deposition causes neuronal damage and impairment in synaptic function, resulting in chronic neurodegeneration, as evident by cognitive impairment, and, eventually, the development of dementia [39]. The aggregation and accumulation of Aβ plaques are postulated as the main cause of Alzheimer’s disease, and there is pathological, biochemical, and genetic evidence to support the amyloid cascade hypothesis. Evidence from clinics and laboratories globally supports the position that the disparity between production and clearance of Aβ peptides such as Aβ_42_ is the initiating factor in the pathogenesis of Alzheimer’s disease [40].

Amyloid beta (Aβ) plaques found in the brains of persons with Alzheimer’s disease consist of Aβ peptides, their main protein component. The Aβ peptides are generated in the central nervous system and comprise of 37–49 amino acid residues derived from the proteolytic cleavage of amyloid precursor protein (APP), a transmembrane protein by β-secretase and γ-secretase [38]. Moreover, Aβ peptides circulate in brain interstitial fluid, CSF, and plasma. The most prevalent peptide isoform present in the brain in physiological conditions is Aβ_40_ (approximately 80–90%), followed by Aβ_42_ (approximately 5–19%). Vascular amyloid consists predominantly of Aβ_40_, while senile plaques contain both Aβ_42_ and Aβ_40_ [41]. The former is fibrillogenic, moves at a faster rate, and is regarded as more hydrophobic in nature [42].

### 3.2. Cerebrospinal Fluid Aβ42 Peptide

The peptide Aβ_42_ contributes significantly to amyloid angiopathy in patients with Alzheimer’s disease [43]. It is the Aβ peptide that is secreted from the neurons in the highest amount, although this is dependent on synaptic activity [44]. Aβ_42_ in cerebrospinal fluid, in combination with p-tau and t-tau, is usually the globally accepted signature for the diagnosis of Alzheimer’s disease [45]. As one of the biomarkers of neurodegeneration in patients with Alzheimer’s disease, its levels in CSF are found to be decreased. Decreased concentrations of CSF Aβ_42_ to approximately 50% have been reported in patients with Alzheimer’s disease compared to healthy controls [46]. In a meta-analysis that comprised 231 studies, including 13,018 controls and 15,699 patients with Alzheimer’s disease, lower CSF Aβ_42_ concentrations discriminated between those with the disease and controls (OR: 0.56, 95% CI: 0.55–0.58, *p* < 0·0001) [27] (Table 1). Likewise, [47] conducted a cross-sectional retrospective evaluation of 17 meta-analyses studies, where there was a comparison of baseline CSF Aβ_42_ concentrations in controls and Alzheimer’s patients. They reported a reduction in 14 studies (2 studies were unclear) [48,49]. However, in one of the studies, there was an increase in Aβ_42_ concentrations in patients with Alzheimer’s disease, particularly in the early and mid-stages [50]. In terms of the clinical utility of CSF Aβ_42_, a mean specificity of 82% (95% CI: 74–88%) and sensitivity of 80% (95% CI: 73–85%) has been stated [51].

The biomarker CSF Aβ_42_, in combination with p-tau and t-tau, establishes acceptable biomarkers for the diagnosis of Alzheimer’s disease. CSF Aβ_42_ is useful for differentiating between Alzheimer’s disease and other neurodegenerative diseases [52,53]. In a prospective cohort study, including 65 patients with Alzheimer’s disease, 26 patients with vascular dementia, and 50 controls, there were significant differences in Aβ_42_ between the two patient groups [53]. In another study, CSF Aβ_42_ had the potential to differentiate Alzheimer’s disease dementia from frontotemporal dementia (sensitivity 85%, specificity 77%) [54]. However, there are studies that have reported that Aβ_42_ was not able to discriminate between Alzheimer’s disease and vascular dementia as its levels were decreased in both disease states [55,56].

There is evidence that suggests that Aβ_42_ may be predictive of the procession of persons with normal cognition and those with mild cognitive impairment [28,57,58]. Buchhave et al. conducted a clinical study of 137 patients with mild cognitive impairment, followed-up for 4.1–11.8 years. They found that that CSF Aβ_42_ precisely predicts the progression of the disease as its levels were significantly reduced at least 5–10 years before conversion to Alzheimer’s disease [57]. Likewise, in the meta-analyses conducted by Diniz and colleagues, low levels of CSF Aβ_42_ were predictive of individuals with mild cognitive impairment who later develop Alzheimer’s disease [28] (Table 1). There was also another study of 44 patients with mild cognitive impairment, where there was a significant decline of CSF Aβ_42_ between baseline and follow-up assessments [58]. Interestingly, an earlier study demonstrated that patients with mild cognitive impairment and lower levels of CSF Aβ_42_ had a faster progression to Alzheimer’s disease [59].

### 3.3. Cerebrospinal Fluid Aβ40 Peptide

Amyloid β1-40 (Aβ_40_) consists of 40 amino acid long residues and is formed due to the imprecise cleavage of the Aβ sequence by γ-secretase, particularly at the C-terminus [60]. The levels of Aβ_40_ in the CSF of normal individuals are significantly higher than that of Aβ_42_ and can be used as a “proxy” for total levels of Aβ. Aβ_40_ is also the predominant isoform present in plasma and brain interstitial fluid and is the main constituent of amyloid plaques in the brains of patients with Alzheimer’s disease and cerebral amyloid angiopathy [61,62].

It is suggested that the interaction between Aβ_40_ and Aβ_42_ has a major role in the development of Alzheimer’s disease. Using electron paramagnetic resonance, Gu et al. demonstrated that Aβ_40_ and Aβ_42_ form in-vitro mixed amyloid fibrils in an interlocked fashion, even though Aβ_40_ is not as effective as Aβ_42_ in terms of being integration into Aβ_42_ fibrils [63]. Likewise, there is cross-seeding between Aβ_42_ and Aβ_40_, and Aβ_40_ fibril seeds can stimulate the aggregation of Aβ_42_ in a concentration-dependent way (and vice versa) [64]. Furthermore, Aβ_40_ does not seem to be as pathogenic as Aβ_42_, and its higher levels do not develop an overt amyloid pathology [65]. It may reduce the risk of developing Alzheimer’s disease as it possesses protective properties with respect to amyloid deposition [66]. Therefore, the ratio of Aβ_40_ to Aβ_42_ may be more important than the levels of either peptide [67].

CSF Aβ_40_ has been investigated as a potential biomarker of Alzheimer’s disease. In a study comprising of 1499 patients with Alzheimer’s disease and 672 non-Alzheimer’s disease subjects, Dorey et al. reported that the CSF Aβ_40_ concentration enhanced the interpretation of Aβ_42_ levels and demonstrated superior diagnostic performance when compared with the Aβ_42_/Aβ_40_ ratio [68]. However, in an earlier study, CSF Aβ_40_ levels did not differ between Alzheimer’s disease and non-Alzheimer’s disease patients. The CSF Aβ_42_/Aβ_40_ ratio may contribute an added value in the discrimination between these two groups of patients in the case of intermediate CSF p-tau-181P values in non-Alzheimer’s disease patients [69]. Moreover, CSF Aβ_40_ levels were similar in Alzheimer’s disease patients possessing the apolipoprotein-E (APOE) ε4 allele and controls. Other authors have suggested that the CSF Aβ_40_ level is not useful in supporting the clinical diagnosis of Alzheimer’s disease [70]. In addition, a meta-analysis comprising of 25 Alzheimer’s disease cohorts and 24 controls demonstrated that Aβ_40_ had a marginal size effect and, therefore, only a minor association [27].

Research has investigated the use of Aβ_40_ in assessing neuropathological changes in Alzheimer’s disease patients [71,72]. Baiardi et al. found no significant variations in the concentrations of CSF Aβ_40_ in Alzheimer’s disease patients and no association with amyloid deposits in the brain. However, the CSF Aβ_42_/Aβ_40_ ratio was a better predictor of Alzheimer’s disease pathology than CSF Aβ_40_ in rapidly progressive dementias [72]. There is also a report of a relationship between CSF Aβ_40_ levels and 18F-flutemetamol PET (which measures the concentrations of brain amyloid β-protein fibrils). The correlation was more significant in apolipoprotein-E (APOE) ε4 allele-negative individuals compared with APOE ε4-positive subjects [73]. Furthermore, CSF Aβ_40_, along with Aβ_42_ and soluble amyloid precursor protein beta (sAPPβ; a direct product of BACE1 enzymatic activity), may be valuable in evaluating drugs such as verubecestat, a BACE1 (aspartyl protease β-site amyloid precursor protein cleaving enzyme 1) inhibitor that discriminatingly decreases Aβ_42_ and Aβ_40_ in Alzheimer’s disease patients [74].

### 3.4. Diagnostic Accuracy of Cerebrospinal Fluid Aβ_42_/Aβ_40_ and Aβ_38_/Aβ_42_ Ratios

There are challenges in making a differential diagnosis of Alzheimer’s disease using core CSF biomarkers as there are similarities in the patterns and symptoms of the disease with subcortical vascular dementia and dementia with Lewy bodies [75]. In a study of 157 subjects comprising 55 sporadic Alzheimer’s disease patients, 23 non-Alzheimer’s disease patients, 45 with other neurological diseases, and 34 normal controls, Shoji and colleagues found that while Aβ_40_ levels were comparable between the groups, a decrease in Aβ_42_ levels caused a substantial elevation in the Aβ_40_/Aβ _42_ ratio. The diagnostic specificity and sensitivity of the Aβ_40_/Aβ_42_ ratio were 82% and 51%, respectively, and the authors suggested that it may be a useful biological marker of Alzheimer’s disease and in the monitoring of treatment [76]. In another study that compared the accuracy of Aβ_42_ and Aβ_40_ in discriminating patients with Alzheimer’s disease dementia, non-Alzheimer’s disease dementia, and normal subjects, the Aβ_40_/Aβ_42_ ratio categorized more patients correctly than the use of the Aβ_42_ concentration alone. The improved diagnostic accuracy of the Aβ_40_/Aβ_42_ ratio (compared with Aβ_42_ concentration only), although not significant, was demonstrated to be 90% vs. 85% when relating Alzheimer’s disease to non-Alzheimer’s disease and 90.8% vs. 87% when relating Alzheimer’s disease to non-Alzheimer’s disease plus controls [77]. Likewise, in a later study, both Aβ_38_/Aβ _42_ and Aβ_40_/Aβ_42_ ratios were significantly different in Alzheimer’s disease patients; the latter (although not significant) differentiated persons in this group of patients from those with frontotemporal dementia. The Aβ_38_/Aβ_42_ and Aβ_40_/Aβ_42_ ratios had better diagnostic potential than the individual biomarkers in identifying Alzheimer’s disease patients [78].

There are other studies that have utilized various CSF biomarkers to differentiate between Alzheimer’s disease and other forms of dementia [79,80]. In a study conducted by Spies et al., the CSF Aβ_40_/Aβ_42_ ratio increases the differential diagnosis of Alzheimer’s disease from other types of dementia syndromes such as frontotemporal dementia, vascular dementia, and dementia with Lewy bodies. The Aβ_40_/Aβ_42_ ratio in Alzheimer’s disease patients was significantly lower than in the other groups [29] (Table 1). In another study where the CSF levels of Aβ_40_ and Aβ_42_ were measured using an amplified luminescent proximity homogenous immunoassay, the CSF Aβ_40_/Aβ_42_ ratio improved the discrimination of Alzheimer’s disease from Parkinson’s disease with dementia and dementia with Lewy bodies, compared with either Aβ_40_ or Aβ_42_ individually [81]. 

The Aβ_40_/Aβ_42_ ratio improved the diagnostic presentation of Aβ_42_ in differentiating Alzheimer’s disease from frontotemporal dementia, dementia with Lewy bodies, and vascular dementia [30] (Table 1). Similarly, CSF Aβ_42_/Aβ_38_ and Aβ_42_/Aβ_40_ ratios displayed better diagnostic accuracy than CSF Aβ_42_ in identifying brain amyloid deposition in prodromal Alzheimer’s disease. These ratios also performed better at distinguishing Alzheimer’s disease from other types of dementia such as Parkinson’s disease dementia, subcortical vascular dementia, and dementia with Lewy bodies [82]. Moreover, the Aβ_42_/Aβ_40_ ratio is stable between the prodromal and demented stages in patients with dementia with Lewy bodies in contrast to what was observed in patients with Alzheimer’s disease. The authors have suggested that the Aβ_42_/Aβ_40_ ratio is extremely useful for distinguishing Alzheimer’s disease patients from those with dementia with Lewy bodies, mainly at the prodromal stage when clinical diagnosis is challenging [83].

### 3.5. Beta-Site Amyloid Precursor Protein Cleaving Enzyme

The production of the various monomeric forms of amyloid-β, comprising of Aβ_42_ and the initiation of the amyloid cascade, is due to the activity of beta-site amyloid precursor protein cleaving enzyme (BACE1) [84]. BACE1 plays a critical role in the pathophysiology of Alzheimer’s disease, and its rates of activity and concentrations have been found to be elevated in body fluids (such as plasma and CSF) and in the brains of Alzheimer’s disease patients [85,86,87]. Fukumota et al. reported significantly increased BACE activity and concentration in the temporal neocortex (63% and 15%, respectively) and frontal neocortex (13% and 14%, respectively) in the brains of Alzheimer’s disease patients that develop amyloid plaques [88]. In another study, there was a 2.7-fold elevation of BACE protein expression in the cortex of the brains of Alzheimer’s disease patients compared to age-matched controls [89].

There are a number of clinical studies that have reported a respectable diagnostic performance of CSF BACE1 activity and concentration in differentiating symptomatic Alzheimer’s disease patients from cognitively healthy controls [31,32]. In a case–control and longitudinal study at a specialized memory clinic, where the patients were followed for 3–6 years, CSF BACE1 activity was significantly higher in Alzheimer’s disease patients than in normal control subjects [31] (Table 1). Similarly, BACE1 activity and protein concentration were significantly increased in Alzheimer’s disease patients compared to elderly healthy controls [32] (Table 1). There was also a significant decrease in age-adjusted CSF BACE activity in Alzheimer’s disease patients compared to control subjects [90]. However, in the ADNI cohort study, there was no difference in CSF BACE1 activity in patients with the disease compared to normal elderly controls [91]. Other studies have reported no difference in CSF BACE concentration in sporadic Alzheimer’s disease patients [87,92] and preclinical Alzheimer’s disease [93] compared with healthy elderly subjects.

There are studies that have detected BACE1 in the CSF of patients with mild cognitive impairment [31,94,95]. Zhong et al. reported higher BACE1 activity in patients with mild cognitive impairment compared to Alzheimer disease patients and controls, and elevated BACE1 protein concentration was related to increased risk for Alzheimer’s disease [94]. Similarly, in a case–control and longitudinal follow-up study, higher BACE1 activity was found in subjects with mild cognitive impairment who developed Alzheimer’s disease compared to persons with mild cognitive impairment who remained stable [31]. Similar findings were reported in other studies that showed greater BACE1 activity in amnestic, mild cognitive-impaired patients compared to Alzheimer’s disease patients [96] and mild cognitive-impaired patients due to Alzheimer’s disease compared to healthy controls [97]. Interestingly, Ewers et al. stated that elevated CSF BACE1 activity in both mild cognitive-impaired and Alzheimer’s disease patients is related to the apoE4 genotype and decrease hippocampal volume [98]. Additionally, the overproduction of CSF BACE1 protein concentrations by stressed glial or neurons decreases during the progression to Alzheimer’s disease, reflecting accelerated brain atrophy [95]. It was observed that BACE1 activity was significantly associated with amyloid-beta peptide levels [94,96] and t-tau in mild cognitive impairment subgroups [31], suggesting its involvement in the pathogenesis of Alzheimer’s disease.

Overall, research on CSF BACE1 activity and its protein concentration has given varied results for Alzheimer’s disease patients. Studies have demonstrated greater BACE1 activity and protein concentration in Alzheimer’s disease compared with elderly health controls and patients with mild cognitive impairment compared to controls or Alzheimer’s disease patients. Therefore, although some of the findings are promising, as CSF BACE1 could be used for early diagnosis and progression monitoring of the disease, significant work involving larger populations is needed to accomplish or confirm these findings.

## 4. Tau Pathology in Alzheimer’s Disease

Tau is a major neuronal microtubule-related protein comprising a diversity of fragments and isoforms in the central nervous system [99]. The six highly soluble protein isoforms of tau are formed due to alternate splicing from the gene MAPT (microtubule-associated protein tau) and comprise 352–441 amino acid residues [100]. The full-length tau protein found in the brain has 29 phosphorylated sites, while truncated tau found in CSF has 12 phosphorylated sites [101]. Tau proteins are located in the axons of neurons in the central nervous system, and their chief role is to promote the assembly and stabilization of microtubules in neuronal axons. These functions of tau are inhibited when the protein becomes phosphorylated [102].

Under normal circumstances, the stabilization of microtubules in the exons of the neurons by tau proteins encompasses the dephosphorylation and phosphorylation of the proteins. Phosphorylation of tau proteins can take place at 5 tyrosine sites and 80 serine or threonine sites. Phosphorylation at positions such as Thr 231, Ser 262, Ser 199, Ser 202, and Ser 205 have been associated with pretangles in neuronal processes [103]. It has been suggested that the increased phosphorylation of tau protein is due to an imbalance regulation of dephosphorylation and phosphorylation due to overactivity of cyclin-dependent-like kinase 5 (CDK5) and glycogen synthase kinase-3β [104]. The reduction in the dephosphorylation of tau protein is due to lower activities of protein phosphatase 2A (PP2A) and protein phosphatase 5 (PP5) [105]. Significantly decreased expressions of PP2A were found in the brains of Alzheimer’s disease patients [106].

The abnormal hyperphosphorylated tau protein loses its ability to promote microtubule assembly, with alteration in the cytoskeleton of neurons and the axonal transport system. There is a destabilization of microtubules, and the abnormal tau proteins are polymerized into paired helical filaments. Their subsequent accumulation in the axon of neurons results in the formation of neurofibrillary tangles [107]. The neurofibrillary tangles or neuropil threads, formed in the allocortex of the medial temporal lobe, spreading to the associative isocortex, cause the undernourishment of neurons and, eventually, neuronal death [108]. It has been reported that Aβ oligomers promote the destabilization of microtubules, and the resulting neurotransmitter deficit and axonopathy observed have been implicated in the development of Alzheimer’s disease [109].

### 4.1. Cerebrospinal Fluid Biomarkers of Tau Pathology

#### 4.1.1. Cerebrospinal Fluid Total Tau (T-Tau)

CSF t-tau and phosphorylated tau (positioned at threonine 181), denoted as t-tau and p-tau, have been comprehensively studied as general biomarkers of neuronal injury in neurodegeneration [110]. CSF t-tau and t-tau, combined with CSF Aβ_42_, are essential biomarkers used in the diagnosis of Alzheimer’s disease [111]. In 2011, the National Institute on Aging and the Alzheimer’s Association issued research criteria that comprised a combination of clinical features and biomarkers such as t-tau and p-tau for the diagnosis of mild cognitive impairment due to Alzheimer’s disease [112].

CSF t-tau is a biomarker that is useful in assessing the extent of neuronal damage and neurodegeneration in Alzheimer’s disease (Figure 2) [113]. In a cross-sectional study of 131 Alzheimer’s disease patients and 72 controls, along with a meta-analysis of 31,333 patients with the disease and 1481 controls, Sunderland et al. found an increase in CSF t-tau concentrations of approximately 300% in most of the studies when values of the biomarker of patients were compared with those of normal controls [47]. In another meta-analysis by Olsson et al. comprising of 13,018 normal controls and 15,699 Alzheimer’s disease patients, CSF t-tau had good diagnostic performance in differentiating both groups (average ratio: 2.54, 95% CI: 2.44–2.64, *p* < 0.0001) [27] (Table 1). Likewise, in a meta-analysis ascertaining the clinical efficacy of biomarkers used to diagnosed Alzheimer’s disease, reported by studies between 1990 and 2010, the sensitivity of t-tau was 82% (95% CI: 76–87%) and its specificity was 90% (95% CI: 86–93%) [51].

Mild cognitive impairment involves an insignificant but noticeable deterioration in cognitive skills, including thinking skills and memory, and is a risk factor for developing Alzheimer’s disease [114]. CSF t-tau has been demonstrated to be a reasonable prognostic indicator of the progression from cognitive impairment to mild cognitive impairment and, subsequently, to Alzheimer’s disease dementia [21]. In another study, elevated CSF t-tau concentrations predicted rapid progression and more aggressive neurodegeneration in patients with mild to moderate Alzheimer’s disease [115]. Likewise, t-tau concentrations were higher in mild cognitive impairment converters compared to normal controls and mild cognitive impairment stable patients. Further, Monge-Argilés et al. noted a sensitivity of 82% (95% CI: 76–86%) and a specificity of 70% (95% CI: 65–75%) for t-tau in differentiating Alzheimer’s disease from mild cognitive impairment [33] (Table 1). However, similar CSF t-tau concentrations were observed in Alzheimer’s disease patients and mild cognitive impairment converters [28,116].

CSF t-tau has been shown to differentiate Alzheimer’s disease patients from non-Alzheimer’s disease patients with dementia with a moderate sensitivity of 78% (95% CI: 72–83%) and a specificity of 75% (95% CI: 68–81) [51]. In a comprehensive meta-analysis, CSF t-tau concentrations in Alzheimer’s disease were higher than in patients with dementia with Lewy Bodies and frontal temporal lobe dementia [34]. The t-tau levels differentiated Alzheimer’s disease from dementia with Lewy bodies with a sensitivity of 73% and a specificity of 90% and from frontal temporal lobe dementia with a sensitivity of 74%. The higher t-tau concentrations in Alzheimer’s disease differentiated it from vascular dementia with a sensitivity of 73% and a specificity of 86% [34] (Table 1). However, the results of these studies indicate that elevated levels of CSF t-tau is not specific to Alzheimer’s disease, and whilst levels vary in other neurodegenerative diseases, extremely high values are observed in Creutzfeldt-Jacob disease compared with Alzheimer’s disease (an increase of at least 10-fold) [117].

#### 4.1.2. Cerebrospinal Fluid Phosphorylated Tau (P-Tau)

CSF p-tau is a biomarker of the deposition of tau in the development of Alzheimer’s disease and may be normal or only mildly elevated in the majority of other neurological diseases [118]. Phosphorylation of amino acid residues in the tau protein is mainly at threonine 181 (p-tau181), which is the main p-tau species that increases very early in the continuum of Alzheimer’s disease [119]. There is the phosphorylation of amino acid residues such as threonine 231 and serine 231 and 199, and CSF p-tau231P may act as an alternate biomarker of neurofibrillary pathology in Alzheimer’s disease [120]. Compared with CSF t-tau, p-tau is more specific to Alzheimer’s disease, and the aggregation of hyperphosphorylated tau molecules produces neurofibrillary tangles [121].

In the development of Alzheimer’s disease, elevated hyperphosphorylated tau and its deposition is a better indication of disease progression and has a stronger relationship with a decline in cognition than Aβ_42_ or Aβ_40_ [122,123]. Additionally, in a meta-analysis by Olsson et al., p-tau levels were strongly related with mild cognitive impairment due to Alzheimer’s disease, with a diagnostic performance of 1.88 (95% CI: 1.79–1.97, *p* < 0.0001) [27] (Table 1). However, although p-tau levels are high in the brain of Alzheimer’s disease patients, CSF p-tau levels were found to be weakly associated with the pathological changes indicative of neurofibrillary tangles in the brain of Alzheimer’s disease patients [120,124].

#### 4.1.3. Diagnostic Utility of Cerebrospinal Fluid Aβ Peptides, T-Tau, and P-Tau Ratios

The development of Alzheimer’s disease occurs over a prodromal period of 20 years, and it has been challenging to find CSF and blood biomarkers that reliably indicate early-stage disease before the occurrence of quantifiable cognitive impairments [125]. Fagan et al. examined the ability of CSF biomarkers to discriminate early-stage Alzheimer’s disease from nondemented aging and their capacity to predict cognitive decline in normal individuals. Increased levels of CSF t-tau and p-tau-181, along with decreased levels of CSF Aβ_42_, were found in persons with very mild or mild Alzheimer’s disease. The CSF t-tau/Aβ_42_ and p-tau-181/Aβ_42_ ratios were better at predicting future dementia in cognitively normal older adults than the individual CSF biomarkers [126]. In a meta-analysis of prospective studies that examined the performance of CSF biomarkers in predicting the conversion from mild cognitive impairment to Alzheimer’s disease, the Aβ_42_/p-tau ratio exhibited a high predictive value, particularly in patients younger than 70 years [21]. Likewise, in another study of 137 patients with mild cognitive impairment, followed clinically for 4–6 years, and 39 healthy individuals with normal cognitive function, the combination of Aβ_42_ and t-tau had a sensitivity of 95% and a specificity of 87%. The authors suggested the findings were strongly related to future incipient Alzheimer’s disease in patients with mild cognitive impairment [127]. Conversely, in a meta-analysis involving 5 prospective studies (140 cases and 290 noncases), the CSF P-tau/Aβ_42_ ratio had sensitivities ranging from 80% to 96% and specificities between 33% and 95%. The authors indicated that the variations in sensitivities and specificities could be due to participation sampling, recruitment sources, and reference criteria for target disorders [128]. 

Cerebrospinal fluid tau (total and phosphorylated) and amyloid-β have been extensively studied as individual biomarkers, and their combination has improved their diagnostic performance for identifying patients with Alzheimer’s disease [35,129]. Hulstaert et al. reported that combined assessment with CSF Aβ_42_ and tau significantly improved the discrimination of Alzheimer’s disease patients from the controls and other neurologic disorders (sensitivity of 85% and specificity of 86%) [35] (Table 1). Moreover, in a meta-analysis of studies examining the diagnostic accuracy of CSF t-tau, p-tau, and Aβ_42_ in discriminating between frontotemporal lobar degeneration dementias and Alzheimer’s disease, the p-tau/Aβ_42_ ratio had the best distinguishing value, especially in more cognitive-impaired patients [129].

The pathology involved in the development of Alzheimer’s disease comprises increased levels of t-tau and p-tau in the CSF due to their release from injured and dying neurons and the polymerization of soluble Aβ to form insoluble plaques in the brain [130]. Fagan investigated whether CSF biomarkers in combination were able to detect underlying pathologies such as neurofibrillary tangles or amyloid plaques in individuals with normal cognitive function or mild-to-moderate Alzheimer’s disease dementia. The p-tau-181/Aβ_42_ ratio and Aβ_42_ levels had the best diagnostic performances in detecting persons with underlying amyloid plaque pathology [131]. Moreover, in a study involving the combination of CSF amyloid-β and tau biomarkers in the Oxford Project to Investigate Memory and Ageing (OPTIMA) cohort, higher CSF t-tau and p-tau and lower CSF Aβ_42_ were found in Alzheimer’s disease patients. CSF p-tau/Aβ_42_ (sensitivity of 94% and specificity of 90%) and t-tau/Aβ_40_ ratios (sensitivity of 92% and specificity of 94%) were high discriminators of autopsy-confirmed Alzheimer’s disease from controls. Notably, p-tau/Aβ_42_ (sensitivity of 88% and specificity of 100%) was a good discriminator of autopsy-confirmed Alzheimer’s disease from other dementia syndromes [36] (Table 1). Finally, Shaw et al. sought to develop a CSF biomarker signature using t-tau, p-tau, and Aβ_42_ in Alzheimer’s disease neuroimaging initiative individuals (ADNI). The t-tau/Aβ_42_ ratio identified 89% of ADNI subjects with mild cognitive impairment and predicted their conversion to Alzheimer’s disease [132].

## 5. Vascular Dysregulation in Alzheimer’s Disease

Alzheimer’s disease is characterized by advanced and incapacitating dementia in aging individuals, and the pathogenesis of the disease involves an irregular buildup of tau proteins and amyloid-beta peptides in the neurons and extracellular space of particular regions of the brain [133]. Vascular dysregulation has been linked to the initial pathological event that precedes amyloid-beta peptides and is a major causative factor to dementia and cognitive impairment observed in patients with Alzheimer’s disease [134]. Multifactorial mechanisms involving abnormalities in CSF and plasma biomarkers such as t-tau and p-tau and spatiotemporal alterations in brain amyloid-beta peptide deposition from the ADNI cohort suggest that vascular dysregulation is the strongest and earliest pathological event concomitant with late-onset Alzheimer’s disease [135].

Significant evidence suggests that vascular dysregulation diminishes oxygen, glucose, and other important nutrients to the brain, resulting in damage to parenchymal cells and blood–brain barrier dysfunction, with subsequent indirect neurotoxic effects such as inflammation, dysregulation of nitric oxide, oxidative stress, and paracellular permeability [136]. The reduced cerebral blood flow and hypoxic conditions increase the buildup of amyloid-β peptides in the brain via the stimulation of transcription of the β-secretase 1 (BACE1) gene and the γ-secretase complex [137]. Furthermore, blood–brain barrier disruption initiates cerebral microangiopathy and reduces the clearance of amyloid-beta peptides from the brain [138].

### Cerebrospinal Fluid Heart-Type Fatty Acid Binding Protein (hFABP)

There is a minimum of three fatty-acid-binding proteins (FABPs) located in the human brain, namely, brain-type (b)-FABP, heart-type (h)-FABP, and epidermal-type (e)-FABP. Heart-type fatty-acid-binding protein (hFABP) is a protein of molecular weight of 25kDa, present in the cytoplasm of cardiac muscle cells. It is secreted after an ischemic event and is regarded as an early biomarker for myocardial infarction [139]. Iturria-Medina et al. conducted a spatiotemporal analysis of a number of CSF and plasma analytes from the ADNI cohort and found abnormally high levels of hFABP that were associated with vascular dysfunction [135].

Basic experimental and epidemiological research has reported a relationship between dyslipidemia and the development of Alzheimer’s disease [140,141]. Heart-type fatty-acid-binding protein is involved with the transportation of lipids and fatty acid metabolism and has been implicated as a biomarker of brain atrophy in patients with Alzheimer’s disease [142]. Heart-type fatty-acid-binding proteins could be intricately involved in neurological and cellular dysfunction, and decreased levels have been observed in the brain of Alzheimer’s disease patients [143]. An earlier study by Guo et al., comprising 69 patients with Alzheimer’s disease dementia and 92 healthy controls, found that heart-type fatty-acid-binding protein discriminated between patients with Alzheimer’s disease dementia and healthy controls, with a sensitivity of 57% and a specificity of 76% [144]. Conversely, in the meta-analysis by [27], using data from two clinical studies comprising healthy controls and Alzheimer’s disease patients, there was no relationship between the groups when serum hFABP was used as the biomarker for assessing vascular dysregulation.

There are studies suggesting that CSF concentrations of hFABP may have promising diagnostic and prognostic potential in preclinical Alzheimer’s disease [145]. CSF hFABP levels begin to elevate at the very early stages of Alzheimer’s disease, suggesting that this biomarker may be a good predictor of the disease [146]. The ADNI cohort study evaluated CSF and plasma novel biomarkers in detecting Alzheimer’s disease at its prodromal stage. An amalgamation of CSF hFABP, tumor necrosis factor-related apoptosis-inducing ligand receptor 3 (TRAIL-R3), cortisol levels in plasma, and fibroblast growth factor 4 permitted a consistent prediction of the disease. The diagnostic accuracy of the combination of these biomarkers had a sensitivity of 88% and specificity of 70% [146]. Likewise, CSF hFABP predicted the progression from mild cognitive impairment to Alzheimer’s disease dementia. Its combination with established markers such as p-tau-181, t-tau, and Aβ_42_ has greater overall diagnostic accuracy for assessing the different stages of Alzheimer’s disease [144] (Table 2).

There is a need for novel CSF and plasma biomarkers to improve the prognostic and differential diagnosis of Alzheimer’s disease with an acceptable discriminating potential for this condition compared with other neurodegenerative diseases such as vascular dementia, Parkinson’s disease, dementia with Lewy bodies, and frontotemporal dementia [147]. In a study that investigated the diagnostic performance of five CSF biomarkers in a well-characterized cohort of patients with other neurodegenerative diseases, levels of hFABP 3 were significantly elevated in Alzheimer’s disease patients compared with those with Parkinson’s disease. The combination of CSF hFABP 3 with p-tau improved the diagnostic accuracy for differentiating between Alzheimer’s disease and dementia with Lewy bodies [148] (Table 2). In an earlier study, elevated serum hFABP was observed in patients with dementia with Lewy bodies and Parkinson’s disease dementia compared with Alzheimer’s disease [149]. Serum hFABP/CSF tau protein levels better differentiated between Alzheimer’s disease and dementia with Lewy bodies [149].

**Table 2:** Pathological mechanisms (vascular dysregulation and neuroinflammation) and associated biomarkers of Alzheimer’s disease and the findings of related studies.

Pathological Mechanism	Biomarker	Study Design Cohort-Participants	Main Findings	References
Vascular dysregulation	hFABP	49 patients with MCI, 69 patients with AD dementia, and 92 controls	hFABP and vascular endothelial growth factor, Aβ_42,_ t-tau, and p-tau-181 (sensitivity 83% and specificity 86%) distinguish AD from others; hFABP predicted the progression from MCI to AD dementia	[144]
Vascular dysregulation	hFABP	208 patients enrolled in 3 European centers (48 AD, 40 DLB, 20 PDD, 54PD)	FABP3 with p-tau showed high accuracy for differential diagnosis between AD and DLB (AUC 0.92)	[148]
Neuroinflammation	TREM2 (R47H substitution)	Case–control series of 2261 subjects with identified sequence variants (AD and controls)	R47H substitution conferred a significant risk of Alzheimer’s disease (OR: 2.92; 95% CI: 2.09–4.09; *p* = 3.42 × 10^−10^)	[150]
Neuroinflammation	TREM2 (R47H substitution)	1092 patients with Alzheimer’s disease and 1107 controls	Highly significant association (OR: 5.05, 95% CI: 2.77–9.16; *p* < 0.001)	[151]
Neuroinflammation	sTREM2	AD case–control dataset (n = 180) and 40 TREM2-risk variant carriers	AD cases presented higher CSF sTREM2 levels vs. controls (*p* = 0.01); CSF sTREM2 levels significantly higher in R47H carriers vs. non-carriers (*p* = 6 × 10^−3^)	[152]
Neuroinflammation	YKL-40	37 cognitively normal, 61 MCI, and 65 AD patients from a memory-clinic-based Amsterdam dementia cohort	Baseline levels of YKL-40 higher in MCI and AD patients and predicted progression to AD (HR 95% CI: 3.0 (1.1–7.9))	[153]
Neuroinflammation	Osteopontin	67 AD patients, 46 FTD patients, and 69 controls	Osteopontin levels were significantly increased in AD patients, correlated with the MMSE score, and were higher in the early disease phases	[154]
Neuroinflammation	Osteopontin	35 AD patients, 31 MCI patients, and 20 other noninflammatory neurologic diseases (ONDs)	Osteopontin levels significantly increased in AD and MCI converters compared to OND	[155]
Neuroinflammation	GFAP	27 AD patients, 13 young controls, 9 adult controls, and 8 senescent controls	GFAP level of AD (8.96 ± 7.80 ng/mL) significantly higher than all-controls (3.19 ± 1.39 ng/mL, *p* < 0.001) and age-matched senescent (3.99 ± 1.55 ng/mL, *p* < 0.005)	[156]
Neuroinflammation	MCP-1	30 controls and 119 patients with MCI (followed for 5 years); 47 developed AD	MCP-1 levels were significantly elevated in prodromal AD patients compared to controls and correlated with a faster cognitive decline and development of dementia	[157]
Neuroinflammation	Progranulin	Progranulin determined in patients in the Dominant Inherited Alzheimer’s Disease Network (DIAN) and the Alzheimer’s Disease Neuroimaging Initiative (ADNI)	Progranulin increased over the course of the disease and significantly differed from noncarriers; in late-onset AD, higher levels were associated with more advanced disease stages and cognitive impairment	[158]

## 6. Inflammation and Glial Activation in Alzheimer’s Disease

### 6.1. Cerebrospinal Fluid Triggering Receptor Expressed on Myeloid Cells

Triggering receptor expressed on myeloid cells 2 (TREM-2) is a 230 amino acid cell surface transmembrane glycoprotein encoded by the TREM2 gene located on human chromosome 6p21 [159]. TREM-2 is expressed by microglia in the brain, and its expression differs based on the specific region of the central nervous system [160]. The ectodomain of TREM-2 is proteolytically cleaved, secreted into extracellular space, and detected in CSF [161]. TREM-2 possesses either inhibitory or activating functions, and its expression is enhanced by anti-inflammatory molecules or decreased by proinflammatory molecules such as interleukin-β, lipopolysaccharides, and tumor necrosis factor-α [162]. It has numerous physiological functions, which include mediating the phagocytosis pathway, modulating inflammatory signaling, and regulating the number, proliferation, and survival of myeloid cells [163].

Homologous mutations in TREM-2 genes have resulted in several genetic variants that have been associated with an increased risk of developing Alzheimer’s disease and other neurodegenerative diseases [164,165]. Several rare variants in TREM-2 have been identified to substantially increase the risk of developing late-onset Alzheimer’s disease by 2–4 times compared to the increased risk related to one copy of the ε4 allele of the APOE gene [166]. A meta-analysis performed by Guerreiro et al. on data from 1092 patients with Alzheimer’s disease and 1107 controls found that the most common variant in the patients was rs75932628 (encoding R47H) [151]. The variant rs75932628 is a single nucleotide polymorphism in the TREM gene located on chromosome 6, causing an R47H substitution [167]. The variant rs75932628 confers a substantially higher risk of late-onset Alzheimer’s disease, and, in one study, the odds ratio was 2.9 [150] (Table 2); in another study, which comprised 1887 patients with Alzheimer’s disease, a strong, highly significant association (OR: 5.05, 95% CI: 2.77–9.16; *p* < 0.001) was observed [151] (Table 2). In another study conducted in a Caucasian population comprising 4567 late-onset Alzheimer cases, carriers of the R47H variant had a significantly higher risk (OR: 7.40, *p* < 0.001) [168].

There are other studies that have confirmed an association between the TREM-2 R47H mutation and the risk of Alzheimer’s disease [169,170]; it is noteworthy that a recent study suggested the possibility that an APOE4 allele in R47H mutation carriers may be essential for Alzheimer’s disease to be developed in these individuals [171]. However, in a study of 2190 late-onset Alzheimer’s disease patients, no association was observed between the TREM-2 R47H mutation and Alzheimer’s disease [172]. The R47H TREM-2 variant has shown a significant association with global decline and tau pathology [173] and increased neurofibrillary tangles and density of amyloid plaques in multiple brain regions, as well as soluble TREM-2 (sTREM-2) levels [174]. Other TREM-2 variants associated with the risk of Alzheimer’s disease have been identified and include rs72824905, rs616338, and rs143332484 [175].

The neuropathology of Alzheimer’s disease is related to neuroinflammation, and studies have been conducted to investigate the diagnostic potential of plasma and CSF sTREM-2 as a biomarker for microglia activity in early-stage Alzheimer’s disease [176]. In a study by Heslegrave et al., the levels of CSF sTREM-2 were significantly higher in Alzheimer’s disease patients compared to cognitively normal individuals, indicating that this biomarker is able to quantify glial activation in the disease. The authors also reported significant relationships between CSF sTREM-2 and biomarkers of neurodegeneration, such as p-tau-181 and t-tau [177]. In a case–control study by [152], which included 180 Alzheimer’s disease cases and 40 TREM-2 risk variant carriers, higher CSF sTREM-2 levels were observed in patients with the disease than controls and also in R47H carriers compared to noncarriers [152]. However, in a prospective cohort study, no significant difference in the levels of CSF sTREM-2 was observed between controls and Alzheimer’s disease patients. Notably, CSF sTREM-2 was correlated positively with core neurodegenerative biomarkers such as Aβ42, p-tau, and t-tau [178]. Furthermore, while the levels of CSF sTREM-2 in Alzheimer’s disease were significantly higher compared with control, plasma sTREM-2 levels showed no significant differences between the groups [179].

### 6.2. Cerebrospinal Fluid Chitinase-3-Like Protein 1

YKL-40, commonly known as chitinase-3-like protein-1, is a 40-kDa mammalian glycoprotein with a sequence similar to family 18 of fungal and bacterial chitinases [180]. YKL-40 is a biomarker that is upregulated in a number of inflammatory disorders and solid tumors, and elevated levels are concomitant with poor prognosis and survival in patients with cancer [181,182]. It is synthesized and expressed by immune and nonimmune cells such as neutrophils, macrophages, endothelial cell fibroblasts, and vascular smooth muscles, and it is activated by cytokines such as tumor necrosis factor-α, interleukin-1β, interferon-γ, and interleukin-13 [183]. In neurodegenerative disorders such as Alzheimer’s disease, YKL-40 is expressed in microglia and astrocytes close to Aβ plaques and is positively associated with t-tau and p-tau, signifying a key role for YKL-40 in the inflammatory processes of Alzheimer’s disease [184,185].

Studies have proposed the potential value of determining CSF YKL-40 levels in the diagnosis of Alzheimer’s disease [186,187]. Janelidze et al., using the longitudinal Swedish BioFINDER cohort, investigated the association of YKL-40 that reflects neuroinflammation and astrocyte and microglia activation with core biomarkers of Alzheimer’s disease [186]. CSF YKL-40 levels were elevated during the preclinical, prodromal, and dementia stages of Alzheimer’s disease and concomitant with high CSF t-tau and p-tau protein levels, particularly for Aβ-positive individuals. It was observed that higher levels of CSF YKL-40 increased the risk of progression of Alzheimer’s disease dementia in nondementia patients [186]. Similarly, Antonell et al. reported significantly high levels of CSF YKL-40 in patients at the prodromal phase when compared with controls with cognitively normal function. Of note is a significant correlation of CSF YKL-40 with p-tau and t-tau proteins in the preclinical Alzheimer’s disease group [187]. The results of two other studies are in agreement with these observations. In a review, CSF YKL-40 levels were significantly elevated in Alzheimer’s disease patients compared to cognitively normal controls and correlated with amyloid-beta peptides, t-tau, and p-tau proteins [188]. In an earlier study, CSF YKL-40 levels of Alzheimer’s disease patients were about twice higher than healthy controls with normal cognitive function [189]. Likewise, higher mean CSF YKL-40 levels were observed in very mild and mild dementia patients (Clinical Dementia Rating 0.5 and 1) compared with cognitively normal subjects. Importantly, the authors found that the CSF YKL-40/Aβ42 ratio was similar to t-tau/Aβ_42_ and p-tau-181/Aβ_42_ ratios in predicting the risk of developing cognitive impairment [184].

Neuropathological trademarks of Alzheimer’s disease include synaptic degeneration and neuroinflammation, and studies have investigated biomarkers such as YKL-40 that assess microglial activation and synaptic damage in the course of the disease [153,188,190]. Kester et al. examined the clinical utility of CSF YKL-40 levels of participants from the memory-clinic-based Amsterdam Dementia Cohort, with a mean cognitive follow-up of approximately 4 years [153]. CSF YKL-40 levels were higher in Alzheimer’s disease and mild cognitive-impaired patients than in persons with normal cognitive function. Baseline values in the mild cognitive impairment group predicted progression to symptomatic Alzheimer’s disease. Additionally, CSF YKL-40 levels may be associated with the progression of the disease as it increased longitudinally in mild cognitive impairment and Alzheimer’s disease patients but not in persons with normal cognitive function [153] (Table 2). In a recent pilot study, longitudinal records demonstrated an elevation in CSF YKL-40 levels in mild cognitive impairment patients as they approach symptomatic Alzheimer’s disease [191]. These observations were confirmed in a study of the ADNI cohort, where, longitudinally, there was an elevation of CSF YKL-40 levels in all the groups over the mean follow-up time of 4 years, although the change in the biomarker was significant only in mild cognitive-impaired Aβ-positive patients [190]. Moreover, greater CSF YKL-40 levels and YKL-40/Aβ_42_ ratios have been concomitant with an elevated risk of progression from normal cognitive function to mild cognitive impairment of participants in a multicenter study [184]. Interestingly, elevated levels of CSF YKL-40 were observed in patients with dementia or mild cognitive impairment due to Alzheimer’s disease, although there was no correlation with core biomarkers such as t-tau and p-tau proteins and Aβ_42_ in Alzheimer’s disease patients [192]. Furthermore, the determination of CSF YKL-40 levels may differ between patients with mild cognitive impairment in a stable phase from those who have progressed to Alzheimer’s dementia [188].

YKL-40, as a biomarker of neuroinflammation and activation of microglia, has been assessed in a number of neurological disorders, including Alzheimer’s disease, where the pathophysiology involves pathological deposits of atypical proteins such as tau proteins, Aβ peptides, and alpha-synuclein, amongst others [193]. Alcolea et al. investigated the diagnostic utility of CSF YKL-40 in a large cohort of patients with Parkinsonian syndromes and different dementias. High levels of CSF YKL-40 were observed in patients with Alzheimer’s disease and frontotemporal lobar degeneration [194]. The sAPPβ/YKL-40 ratio had an area under the curve of 0.84 in distinguishing the two groups of patients [194]. In a multicenter study aimed at assessing the diagnostic utility of CSF YKL-40 in differentiating clinical Alzheimer’s disease from other neurodegenerative diseases, the biomarker discriminated Alzheimer’s disease from frontotemporal dementia [195]. In a recent study, levels of CSF YKL-40 were higher in frontotemporal lobar degeneration patients with tau pathology compared with Alzheimer’s disease patients, and the sAPPβ/YKL-40 ratio was sufficiently distinguished between the groups (AUC: 0.70, 95% CI: 0.61–0.79) [196].

Wennstrom et al. evaluated the disease-specificity of CSF YKL-40 levels in patients with dementia with Lewy bodies, Parkinson’s disease, and Alzheimer’s disease. CSF YKL-40 levels in patients with Alzheimer’s disease, after correcting for age, were found to be 27.7% and 38.8% lower in patients with dementia with Lewy bodies and Parkinson’s disease, respectively, compared with Alzheimer’s disease [197]. In a recent study, CSF YKL-40 levels increased in Alzheimer’s disease patients but not in dementia with Lewy bodies, Parkinson’s disease dementia, or vascular dementia [198]. Other studies have shown increased CSF YKL-40 levels in Alzheimer’s disease patients compared with Parkinson’s disease dementia and dementia with Lewy bodies [199], dementia and prodementia with Lewy bodies [53,200], and vascular dementia [53].

In summary, the findings from these studies demonstrate that YKL-40 might be a suitable biomarker for the diagnosis of Alzheimer’s disease. CSF YKL-40 levels are elevated in Alzheimer’s disease and may have value for discriminating subjects with normal cognitive function and patients with Alzheimer’s disease or mild cognitive impairment. There are studies that have established the association of CSF YKL-40 levels with core biomarkers of Alzheimer’s disease such as t-tau and p-tau proteins; increased levels of this biomarker may be related to disease progression. CSF YKL-40 levels may discriminate Alzheimer’s disease from dementia with Lewy bodies, Parkinson’s disease, or vascular dementia. A combination of high levels of CSF YKL-40 with low sAPPβ may have the utility to differentiate Alzheimer’s disease from frontotemporal dementia.

### 6.3. Interferon Gamma-Induced Protein: Pathology and Cerebrospinal Fluid Levels

Interferon gamma-induced protein 10 (IP-10), commonly known as C-X-C motif chemokine 10 (CXCL10), is a small protein of molecular weight 8.7 kDa that is encoded by the gene CXCL10 in humans [201]. During inflammation in response to interferon-γ, interferon-α, and interferon-β, it is secreted by different types of cells, including epithelial, fibroblast, monocyte, leukocyte, stromal and endothelial, keratinocyte, neutrophil, and eosinophil cells [202]. The biological effects of IP-10 are exerted by its binding and activation of a seven transmembrane-spanning G-protein-coupled chemokine receptor, denoted CXCR3. By binding to the carboxyl-terminal region of CXCR3, B- and T-lymphocytes, macrophage and dendritic cells, and natural killer cells are activated [203]. As a pleiotropic molecule, IP-10 induces a number of biological functions, including the regulation of cell growth and apoptosis and the induction of chemotaxis [204].

An important role of IP-10 is regulating the entry of subsets of leukocytes into various tissues as well as the brain; its expressions are usually absent in the resting central nervous system [205]. During neuroinflammation, IP-10 is expressed by glia, stromal cells, and neurons, and its detrimental role in enabling disease progression has been implicated in a number of neurodegenerative disorders [206]. It has been suggested that the induction of IP-10 by astrocytes and microglia and its binding to CXCR3 of neurons may lead to the initiation of the extracellular-signal-regulated kinase pathway, with subsequent neuronal dysfunction and programmed cell death [207].

IP-10 and CXCR3 contribute to the pathophysiological neuroinflammatory process in the development of Alzheimer’s disease by recruiting monocytes and activating astrocytes and glial cells [208]. The upregulation of a number of chemokines and their receptors has been connected with pathological changes observed in Alzheimer’s disease [209]. Xia et al. reported that IP-10 is elevated in astrocytes in Alzheimer’s disease brains and has been associated with senile plaques [210]. Galimberti et al. determined CSF IP-10 levels in subjects with amnestic mild cognitive impairment, Alzheimer’s disease patients, and their age-matched controls. CSF IP-10 levels were significantly increased in patients with mild cognitive impairment and mild Alzheimer’s disease but not in patients with severe Alzheimer’s disease (MMSE score <15), as the biomarker appears to decrease with the progression of the disease [211]. Similar observations were reported in another study by the same group of authors [211]. Moreover, in a study that explored whether CSF biomarkers of neuroinflammation independently predict and provide an indication of the pathology and neuronal damage associated with Alzheimer’s disease, there were elevated levels of IP-10 in patients with the disease compared to controls with normal cognitive function. It was also found that higher CSF IP-10 levels correlated with elevated levels of CSF t-tau [212]. However, in a later study, CSF IP-10 levels were not increased in a group of Alzheimer’s disease patients with mild to moderate dementia compared to controls with normal cognitive function [197]. In addition, there were no associations between CSF IP-10 levels and Aβ pathology or cognitive decline [197].

### 6.4. Cerebrospinal Fluid Osteopontin

Osteopontin is a glycosylated, phosphorylated, and hydrophilic protein produced by a number of cell types, including T-lymphocytes and macrophages [213]. It is intricate in a number of physiological and pathological developments, including oxidative stress, bone mineralization, apoptosis, angiogenesis, immunity, inflammation, tumorigenesis, cell migration, and wound healing [214,215]. Studies have revealed marked increased osteopontin levels in numerous autoimmune and inflammatory conditions such as systemic lupus erythematosus, rheumatoid arthritis, and inflammatory bowel disease [216,217,218], as well as in neurodegenerative diseases such as Parkinson’s disease and multiple sclerosis [219,220].

There are reports of significantly higher levels of CSF osteopontin in Alzheimer’s disease patients compared with control subjects [221,222,223]. This finding was corroborated in a study by Comi et al., who reported increased CSF osteopontin in Alzheimer’s disease patients compared to controls. CSF osteopontin levels were significantly increased in the early stages of the disease and were associated with a decline in cognition [154] (Table 2). Sun et al. found significantly increased levels of CSF osteopontin in subjects with Alzheimer’s disease and subjects with mild cognitive impairment who later developed the disease compared with controls with other noninflammatory neurologic diseases [155]. The elevated levels of CSF osteopontin were associated with a decline in cognition, thus giving insight into the etiopathogenic role of osteopontin in Alzheimer’s disease [155] (Table 2). In an earlier study, Simonsen et al. employed proteomic analysis of cerebrospinal fluid samples and found elevated levels of a phosphorylated C-terminal fragment of osteopontin in mild cognitive-impaired patients who progressed to Alzheimer disease compared to those who stayed stable over the 4–6 years of follow-up, and healthy controls [224]. Moreover, CSF osteopontin discriminated mild cognitive impairment patients from controls, was a good indicator of the disease, and had a moderate association with cognitive decline [225].

Notably, Wung and colleagues found a 41% elevation in the expression of osteopontin in the cytoplasm of pyramidal neurons in the brains of Alzheimer’s disease patients compared to the brains of age-matched controls. Osteopontin expression was associated with an amyloid-beta load, indicating the role of this biomarker in neuronal remyelination and neurodegeneration in Alzheimer’s disease [226].

In summary, higher CSF osteopontin levels are found in Alzheimer’s disease patients and subjects with mild cognitive impairment who progress to the disease. It is a biomarker of the prodromal Alzheimer’s disease stage and is useful in the monitoring of the disease. These findings suggest the participation of osteopontin in the development of Alzheimer’s disease and points to the need for further wide-scale studies to confirm these results.

### 6.5. Cerebrospinal Fluid Glial Fibrillary Acidic Protein

Glial fibrillary acidic protein (GFAP), with molecular weight 50 kDa, encoded by the human GFAP gene found on chromosome 17q21, is a class-III intermediate filament that is expressed by astrocytes in the central nervous system [227,228]. GFAP is an astrocyte-specific biomarker that has a role in cell mobility and migration, proliferation and astrocyte transformation, vesicle transport and autophagy, and astrocyte–neuron interactions [229,230].

GFAP is a recognized indicator of astrogliosis in neuropathological disorders such as Alzheimer’s disease. The severity of Alzheimer’s disease pathology is associated with increased GFAP expression in CSF and the density of reactive astrocytes [231]. There are studies that have reported higher levels of CSF GFAP in Alzheimer’s disease patients compared to healthy control subjects, reflecting the astrocytosis and degeneration of astrocytes [156,232,233,234]. CSF GFAP was also increased in other neurodegenerative diseases such as Lewy body dementias, Parkinson’s disease, frontotemporal dementia, prion disease, and Creutzfeldt-Jakob’s disease [232,233,234,235]. However, in these studies, the levels of CSF GFAP in Alzheimer’s disease patients, when compared with those of other neurodegenerative diseases, were not significantly different and, therefore, of limited diagnostic value [232,233,234,235]. Moreover, there is evidence that higher levels of CSF GFAP are associated with cognitive decline in Alzheimer’s disease patients at early stages of dementia [236] and also correlate with the severity of dementia [156] (Table 2).

In conclusion, GFAP is an astroglial marker, and there are elevated levels of GFAP in the CSF of Alzheimer’s disease patients. It does not possess significant diagnostic clinical utility in differentiating Alzheimer’s disease from other neurodegenerative disorders but shows potential as a biomarker for cognitive decline and monitoring the disease.

### 6.6. Cerebrospinal Fluid Monocyte Chemotactic Protein-1

Monocyte chemotactic protein-1 (MCP-1) or chemokine CCL2 is a proinflammatory signaling protein that modulates the recruitment, migration, and infiltration of immune cells such as macrophages and monocytes to inflammation sites [237]. The interaction of MCP-1 with its receptor CCR2 causes a cascade of cellular activation events, such as cell migration and survival, induction of chemotactic response, deployment of intracellular calcium ions, and increased activity of the synaptic network in neurons of the hippocampus [238,239]. Elevated levels of MCP-1 have been implicated in various neurodegenerative and neuroinflammatory disorders, including Alzheimer’s disease [240].

In a systematic review and meta-analysis involving CSF biomarkers of glial activation, there was only a mild elevation of MCP-1 that did not differentiate between Alzheimer’s disease patients and controls with normal cognitive function [27]. However, in an earlier study, CSF MCP-1 levels were elevated in Alzheimer’s disease patients and subjects with mild cognitive impairment compared to age-matched controls [211]. There was a significant positive correlation between CSF MCP-1 levels and decreased cognitive function [211]. This finding was confirmed in a longitudinal study, where CSF MCP-1 levels were significantly elevated in prodromal Alzheimer’s disease patients when compared to controls and associated with faster cognitive decline and development of dementia within a shorter time period [157] (Table 2). Notably, a combination of CSF MCP-1 with CSF Aβ_42_ levels and p-tau and t-tau proteins could predict the rate of decline and future conversion to Alzheimer’s disease in patients with mild cognitive impairment [157]. There are other studies that have corroborated these findings [241,242] and reported a positive correlation between increased levels of CSF MCP-1, amyloid-β peptides, and p-tau protein levels [242].

The brains of patients with Alzheimer’s disease are characterized by Aβ-peptides and associated chronic neuroinflammation that involves the expression of MCP-1 by monocytes that mediate the process [243]. In another study, MCP-1 was found in reactive microglia and mature senile plaques of brain tissues from Alzheimer’s disease patients. This finding proposes the role of MCP-1 in the maturation of senile plaques [244]. The enzyme isoglutaminyl cyclase is involved in MCP-1-driven neuroinflammation in Alzheimer’s disease, and there is evidence of increased expressions of the enzyme, MCP-1 mRNA, and protein in patients with the disease compared to controls [245].

In summary, CSF MCP-1 levels are found in both early and late stages of Alzheimer’s and are concomitant with an accelerated rate of cognitive decline. Its combination with traditional biomarkers of neurodegeneration, such as CSF Aβ_42_ and t-tau and p-tau protein levels, makes it a potentially useful biomarker for monitoring the progression of the disease.

### 6.7. Cerebrospinal Fluid Progranulin

Progranulin is an extremely well-maintained glycosylated protein encoded by the GRN gene on position 17q21 of the human chromosome. It is expressed in various types of cells, particularly in the central nervous system and peripheral tissues [246]. Progranulin is regarded as a multifunctional protein and has been involved directly (and through its conversion to granulins) in neurodegeneration, neuroinflammation, wound repair, cell growth, neurodevelopment, and, particularly, the regulation of microglial responses and lysosomal function in the central nervous system [247,248]. Missense mutations found in the analysis of the progranulin gene resulted in a loss of functional progranulin due to the degradation of the misfolded protein; the haplotypes are linked with an increased risk of Alzheimer’s disease [249].

Studies have reported that CSF progranulin levels did not differ in Alzheimer’s disease patients or persons with mild cognitive impairment compared to controls with normal cognitive function [250,251]. Similarly, CSF progranulin levels were not significantly different in patients with early-onset Alzheimer’s disease compared to late-onset disease [252]. However, there was a significant relationship between CSF progranulin levels and overall cognition, and reduced amounts were associated with olfactory dysfunction [251]. A large cross-sectional study of patients with familial and late-onset sporadic Alzheimer’s disease in the ADNI and Dominant Inherited Alzheimer’s Disease Network cohorts reported that CSF progranulin increased over the course of the disorder in carriers with a dominant mutation of the GRN gene [158]. The elevated CSF progranulin levels in patients with late-onset disease were related to cognitive impairment and more advanced disease, as well as higher CSF soluble TREM-2 levels compared with controls [158]. Notably, there was an association of CSF progranulin levels with CSF t-tau protein levels, particularly in patients with developed neurodegeneration, pointing to the impact of this biomarker on tau pathology [145,158].

Overall, progranulin is a neuroinflammation-related protein, and genetic variation may contribute to the risk of Alzheimer’s disease. Levels of CSF progranulin are elevated during the development of Alzheimer’s disease and are related to a decline in cognition and neurodegeneration. Further research is needed to investigate its potential for early detection and the assessment of disease severity.

## 7. Synaptic Dysfunction in Alzheimer’s Disease

Disruption in the activity of synapses and the loss of synapses are regarded as early events in the pathogenesis of Alzheimer’s disease that precede the buildup of Aβ deposits in the brain or the clinical expression of the disease [253]. Synaptic loss is evident by the reduction of synaptic proteins in early Alzheimer’s disease and frank atrophy. The extent of synaptic decline in brains at postmortem has been shown to be associated with cognitive function in persons with early Alzheimer’s disease or mild cognitive impairment [254,255]. The overstimulation of extra-synaptic N-methyl-D-aspartate (NMDA) receptors and the associated synaptic redox stress cause an influx of extracellular calcium, which initiates a series of downstream pathways involving Cdk5/dynamin-related protein 1 (Drp1), caspases, and p-tau. This culminates in mitochondrial dysfunction, apoptosis, and synaptic loss and dysfunction [256,257].

### 7.1. Cerebrospinal Fluid Neurogranin

Neurogranin is a postsynaptic neuronal protein comprising of 78 amino acids that regulate the concentration of calmodulin and, thus, the intracellular calcium–calmodulin signaling pathway. It is mainly expressed in dendritic spines and has a role in the signaling pathway of protein kinase C, where phosphorylation of the latter lowers its ability to bind calmodulin [258,259]. Neurogranin in CSF has been suggested as a biomarker of early synaptic loss and degeneration in Alzheimer’s disease and may be a useful predictor of disease progression [260]. Its involvement in pathophysiological pathways related to Alzheimer’s disease proposes that it may be valuable when combined with other established biomarkers for the diagnosis of early disease [261].

Tarawneh et al. examined the diagnostic efficacy of neurogranin levels in a cross-sectional and longitudinal observational study of 207 cognitively normal controls and 95 individuals with early symptomatic Alzheimer’s disease [262]. The CSF neurogranin levels were significantly higher in patients with early symptomatic Alzheimer’s disease, and this differentiated them from controls, with diagnostic utility (0.71; 95% CI: 0.64–0.77) that was similar to other established cerebrospinal biomarkers [262] (Table 3). Similarly, in a longitudinal study of 37 cognitively normal individuals and 65 patients with Alzheimer’s disease within the memory-clinic-based Amsterdam Dementia Cohort, baseline CSF neurogranin levels in patients with the disease were significantly higher than in cognitively normal participants [263]. It is interesting to note that baseline CSF neurogranin levels were highly associated with p-tau-181 and t-tau but not with Aβ_42_ [263]. In another study published in the same year, CSF neurogranin levels were elevated in patients with Alzheimer’s disease and positively related with CSF t-tau protein and negatively with Aβ_42_/Aβ_40_ [264]. A recent meta-analysis demonstrated that CSF neurogranin levels were significantly greater in Alzheimer’s disease patients compared with individuals with normal cognitive function [265]. High levels of CSF neurogranin in neurologically healthy older adults have been found to be associated with older age and lower levels of CSF t-tau and p-tau proteins but not with Aβ_42_ [266].

**Table 3:** Pathological mechanisms (synaptic dysfunction, neuronal injury) and associated biomarkers of Alzheimer’s disease and the findings of related studies.

Pathological Mechanism	Biomarker	Study Design Cohort-Participants	Main Findings	References
Synaptic dysfunction	Neurogranin	A cross-sectional and longitudinal observational study of 95 AD patients and 207 controls	CSF neurogranin levels differentiated patients with early symptomatic AD from controls with comparable diagnostic utility (0.71, 95% CI, 0.64–0.77)	[262]
Synaptic dysfunction	Neurogranin	Retrospective cohort of 331 participants, including 19 controls, 100 AD, 20 FTD, 13 LBD, 21 PD	Median CSF neurogranin levels higher in AD compared to all other disease groups (all *p* < 0.001); t-tau (*p* < 0.001) and p-tau (*p* < 0.001)	[267]
Synaptic dysfunction	SNAP-25	Three separate cohorts of patients	Significantly higher levels of CSF SNAP-25 fragments in Alzheimer’s disease, even in the very early stages; differentiated Alzheimer’s disease from controls with AUC of 0.901 (*p* < 0.0001)	[268]
Synaptic dysfunction	SNAP-25	139 participants from the ADNI database with normal (CN, *n* = 52), stable mild cognitive impairment (sMCI; *n* = 22), progressive MCI (pMCI; *n* = 47), and dementia due to AD (*n* = 18)	CSF SNAP-25 and SNAP-25/Aβ_42_ were increased in patients with pMCI and AD compared with CN, and in pMCI and AD compared with sMC; significantly predicted conversion from MCI to AD	[269]
Synaptic dysfunction	GAP-43	43 healthy controls, 275 AD, and 344 patients with other neurodegenerative diseases	GAP-43 was significantly increased in AD compared to controls and most neurodegenerative diseases; correlated with the magnitude of t-tau and Aβ plaques	[270]
Synaptic dysfunction	GAP-43	47 AD patients, 17 FTD, 16 VaD, and 12 age-matched controls	GAP-43 increased in AD compared to FTD (*p* < 0.01); positive and highly significant correlation with t-tau	[271]
Neuronal injury	VILP-1	33 AD patients and 24 controls; VLP-1 levels measured using ELISA	VILP-1 concentrations were significantly higher in AD patients (365 ± 166 ng/L) compared to controls (244 ± 142.5); correlation with p-tau (*r* = 0.809)	[272]
Neuronal injury	VILIP-1	61 AD patients, 32 DLB patients, and 40 normal controls using commercial ELISA kits to measure VILIP-1	VILIP-1 level had significantly increased in AD patients compared with controls and DLB patients and accurately diagnosed AD; positively t-tau and p-tau-181P	[273]
Neuronal injury	Neurofilament light	CSF neurofilament (NfL) levels were measured in 221 participants from the Australian Imaging, Biomarkers, and Lifestyle Flagship Study of Ageing (AIBL).	NfL levels, as well as NfL/Aβ_42_, were significantly elevated in AD compared to healthy controls (HC, *p* < 0.001); NfL and NfL/Aβ_42_ differentiated AD from HC, with AUC of 0.84 and 0.90, respectively	[274]
α-synuclein pathology	α-Synuclein	Cohort of 225 AD patients and 68 cognitively intact controls	CSF total α-syn significantly increased in the AD group (*p* < 0.0001) compared to controls; sensitivity 85% and specificity of 84% (AUC = 0.88) in distinguishing AD from controls	[275]
α-synuclein pathology	α-Synuclein	Cohort of approximately 400 healthy control, MCI, and AD subjects in the Alzheimer’s Disease Neuroimaging Initiative	CSF α-synuclein levels were significantly higher in the MCI (*p* = 0.005) and AD (*p* < 0.001) groups compared to controls; AUC of sensitivity (65%) and specificity (74%) as a diagnostic marker of AD	[276]
Iron toxicity	Ferritin	PD, AD, and multiple system atrophy (MSA) as well as control subjects	Significant increase in CSF ferritin in AD compared with both PD and age-matched controls	[277]
Iron toxicity	Ferritin	Longitudinal study involving 296 patients (followed for up to 5 years)	Elevated CSF ferritin (>6.2 ng/mL) was associated with accelerated depreciation of CSF Aβ_42_	[278]
Neurodegeneration and neuroinflammation	miRNAs	Profile of miRNAs in CSF from 50 AD patients and 49 controls using TaqMan^®^ arrays	36 miRNAs that discriminate AD from control; linear combinations of 3 and 4 miRNAs have AUC of 0.80–0.82	[279]
Neurodegeneration and neuroinflammation	miRNAs	10 AD patients and 10 patients diagnosed with either vascular dementia, frontotemporal dementia, or dementia with Lewy bodies	Fifty-two miRNAs were detected in CSF in at least nine out of ten patients in both groups; let-7i-5p and miR-15a-5p were significantly upregulated and miR-29c-3p significantly downregulated in AD patients	[280]

Alzheimer’s disease (AD); Lewy bodies (DLB); frontotemporal lobar degeneration (FTLD); vascular dementia (VaD); Parkinson’s disease with dementia (PDD); Parkinson’s disease (PD); Parkinson’s disease with dementia (PDD); mild cognitive impairment (MCI); enzyme-linked immunosorbent assay (ELISA).

Studies have shown elevated levels of CSF neurogranin in patients with mild cognitive impairments and in the predementia stage of Alzheimer’s disease [281,282]. In the ADNI cohort study, CSF neurogranin levels were significantly increased in Alzheimer’s disease patients with dementia and patients with stable mild cognitive impairment and progressive cognitive impairment compared with controls [281]. In another study, CSF neurogranin levels were significantly higher in patients with prodromal Alzheimer’s disease compared to individuals with mild cognitive impairment. The concentration of this biomarker was positively correlated with established axonal biomarkers of injury, such as CSF t-tau and p-tau proteins, whereas there was no correlation to CSF Aβ_42_ [282]. The meta-analysis by Mavroudis et al. found significantly higher levels of CSF neurogranin levels in mild cognitive-impaired patients who developed Alzheimer’s disease compared with stable mild cognitive-impaired patients [265]. However, in a study reported nine years earlier, there were no substantial differences in CSF biomarkers such as neurogranin, t-tau, and p-tau between healthy controls with normal cognitive function and patients with Alzheimer’s disease [260].

The levels of CSF neurogranin is elevated in Alzheimer’s disease dementia and may be specific for the disease [283]. In a prospective study involving 915 patients, CSF neurogranin levels were significantly and specifically elevated in Alzheimer’s disease compared with eight other neurodegenerative diseases, including amyotrophic lateral sclerosis, Parkinson’s disease, and frontotemporal dementia [284]. In an earlier retrospective cohort study comprised of 19 healthy controls with normal cognitive function and 331 individuals with various neurodegenerative diseases, the median CSF neurogranin level was greater in Alzheimer’s disease patients compared with the other disease groups and controls [267] (Table 3). Notably, the CSF neurogranin level was strongly correlated with p-tau and t-tau proteins [267]. In a cross-sectional multicenter study, CSF neurogranin levels were significantly greater in Alzheimer’s disease patients compared with subjects with frontotemporal dementia and healthy controls with normal cognitive function [283]. Furthermore, neurogranin and other synaptic biomarkers such as Rab3 and SNAP25 were found to predict cognitive decline in patients with Alzheimer’s disease and dementia of Lewy bodies. [285].

A number of studies have shown that CSF neurogranin levels can predict the progression of Alzheimer’s disease [262,263]. In the memory-clinic-based Amsterdam Dementia Cohort study, baseline CSF neurogranin levels were greater in patients with mild cognitive impairment who progressed to Alzheimer’s disease and were prognostic of progress from mild cognitive impairment to Alzheimer’s disease [263]. Elevated levels of CSF neurogranin at the mild cognitive impairment stage predicted progression to Alzheimer’s disease dementia (HR: 12.8, 95% CI: 1.6–103.0) [286]. Similarly, elevated baseline CSF neurogranin levels in patients with mild cognitive impairment forecasted a decline in cognition and corroborated significant longitudinal reductions of hippocampal volume at clinical follow-up [281]. Furthermore, CSF neurogranin levels projected future cognitive impairment (adjusted HR: 1.89; 95% CI: 1.29–2.78) in controls with normal cognitive function and predicted a decline in cognition in patients with symptomatic Alzheimer’s disease [262].

Overall, the data show that CSF neurogranin levels are elevated at the initial clinical stage of Alzheimer’s disease and are specific to the disease. It complements established biomarkers such as t-tau and p-tau proteins and may be a valuable addition to the existing panel of Alzheimer’s disease biomarkers. However, further validation in larger clinical studies is needed.

### 7.2. Cerebrospinal Fluid of Synaptosome-Associated Protein 25 (SNAP-25)

Synaptosome-associated protein 25 (SNAP-25) and synaptotagmin are small tail-anchored and transmembrane proteins that mediate the fusion and exocytosis of synaptic vesicles in neurons with the release of neurotransmitters [287]. During membrane fusion and exocytosis, target-cell-associated t-SNARESs and vesicle-associated v-SNAREs assemble to form a core trans-SNARE complex [288]. The core trans-SNARE complex is an established four-helix structure that connects plasma and vesicle membranes and comprises SNAP-25, which participates with two helices and syntaxin and synaptobrevin, each donating one helix [289].

SNAP-25 is encoded by the SNAP gene and is a 25 kDa presynaptic membrane-bound protein found in plasma. It plays a key role in mediating the specificity of fusion and, as part of the SNARE complex, affects the fusion of synaptic vesicles and plasma membranes [290]. This leads to exocytosis and the regulated related release of neurotransmitters, a major stage in neurotransmission that is essential to normal brain function [291]. Syntaxins are multidomain t-SNARE transmembrane proteins with a single aminoterminal cytoplasmic region (consisting of a regulatory domain and a SNARE domain) and a distinct transmembrane domain [292]. There are about 15 types of syntaxins; the 4 expressed in the plasma membrane are involved in the different stages of membrane fusion and calcium-triggered exocytosis [293].

Synaptobrevin is also known as a vesicle-associated membrane protein that has a molecular weight of 19 kDa. It is attached to the synaptic vesicle via a distinct transmembrane domain. As part of the core trans-SNARE complex, it is intricately linked to calcium-dependent exocytotic membrane fusion by the release of neurotransmitters [294]. Synaptotagmin membrane-trafficking calcium-sensing proteins are described by a distinct transmembrane domain at the N-terminus, an adapter linker domain, and two calcium-binding C2 domains, C2A and C2B [295]. There are 17 different synaptotagmin isoforms, which include calcium-binding synaptotagmins 1, 2, 3, 5, 6, 7, 9, and 10. However, in neurons, synaptotagmins 1 and 2 are the main calcium-sensing proteins that promote anionic membrane-binding with a subsequent fusion of the synaptic vesicle with the presynaptic membrane via the SNARE complex [296]. 

Alzheimer’s disease is described by a progressive decrease in cognition, and the pathology of the synapse is important to the clinical presentation of the disease [297]. Studies have assessed the levels of synaptic proteins such as SNAP-25, β, syntaxin, and synaptotagmin in the brains of Alzheimer’s disease patients and in controls. Postmortem investigations on the brains of Alzheimer’s disease patients have revealed different levels of these synaptic proteins, indicating that they are affected by the disease process [298,299,300].

In a study by Sze at al. (2000), synaptobrevins were decreased by 29% in the hippocampus of patients with early Alzheimer’s disease compared to controls. There were also significant reductions in synaptobrevins (46%) in the hippocampus and synaptobrevins (31%) in the entorhinal cortex of Alzheimer’s disease patients compared to controls [301]. There were decreases in synaptophysin and synaptobrevin levels by approximately 30% and 10% in levels of SNAP-25 and synaptotagmin in the brain of Alzheimer’s disease patients compared with controls [302]. In another study, mean values of SNAP-25, syntaxin, and synaptophysin levels were significantly reduced by 21–28% in the prefrontal cortex of Alzheimer’s disease patients compared with controls [303]. Likewise, there were decreased SNAP-5 levels in five brain regions of patients with Alzheimer’s disease [304] and a 25% reduction of synaptophysin levels in the frontal cortex of patients with early Alzheimer’s disease compared with controls [305]. Notably, in the latter study, the level of synaptotagmin was not different between patients with Alzheimer’s disease and controls [305]. In two recent studies worth noting, there were significantly higher levels of SNAP-25 and synaptotagmin-1 in patients with atypical Alzheimer’s disease compared to those with frontotemporal dementia [306], and patients with vascular dementia had lower CSF synaptophysin levels than their counterparts in the Alzheimer’s disease group [307].

Alzheimer’s disease is associated with cognitive impairment, and synaptic degeneration is suggested to be an early event in the pathophysiology of the disease [308]. There are studies that have investigated synaptic biomarkers in CSF and serum as potential tools for early diagnosis and monitoring disease progression [309]. In a recent study by Agliardi et al., it was noted that SNAP-25 in serum is transported by neuron-derived exosomes and its concentration was reduced in patients with Alzheimer’s disease compared with healthy controls. The sensitivity of serum SNAP-25 to discriminate between the two groups was 87.5%, with a specificity of 70.6%, and there was a significant correlation with cognitive decline in Alzheimer’s disease patients [310]. In another study, there were significantly higher levels of serum SNAP-25 in patients with atypical Alzheimer’s disease compared with controls [306]. Moreover, CSF SNAP-25 levels, determined using mass spectrometry, were significantly elevated in early-stage and established Alzheimer’s disease, and the biomarker was able to meaningfully discriminate patients with the disease from healthy controls with an area under the curve of 0.901 [311]. Notably, in a recent study, CSF SNAP-25 levels were significantly elevated in persons carrying autosomal-dominant Alzheimer’s disease mutations. It was observed that the levels of CSF SNAP were altered about 15–19 years prior to the onset of symptoms, indicating that synaptic damage commences just after the accumulation of brain amyloid-β protein [268].

The degeneration of synaptic proteins is a principal event in the development of Alzheimer’s disease that takes place early during the progression of the disease and is associated with cognitive symptoms [297]. In a recent study, CSF SNAP-25 and SNAP-25/Aβ_42_ were higher in patients with progressive mild cognitive impairment and those with dementia due to Alzheimer’s disease compared to normal controls. It was also observed that increased SNAP-25/Aβ_42_ ratios were higher in cognitively normal individuals who had progressed to mild cognitive impairment or Alzheimer’s disease patients during a follow-up [312]. Furthermore, in a study of the ADNI cohort, comprising cognitively normal, mild cognitive impairment, and Alzheimer’s disease patients who were further defined by amyloid-β status, reference point CSF SNAP-25 levels were higher in Alzheimer’s disease patients and those with mild cognitive impairment who were amyloid-β-positive than cognitively normal individuals (amyloid-β-positive or -negative [190]. Noticeably, CSF SNAP-25 levels reduced longitudinally in the group of Alzheimer’s disease patients followed for 4 years [190].

### 7.3. Cerebrospinal Fluid Synaptotagmin

Synaptotagmin is a calcium sensor presynaptic protein that is critical for the preservation of an intact synaptic transmission and cognitive function, and studies have examined whether selective or regional alterations occur in Alzheimer’s disease [269,313]. Sze et al. reported that synaptotagmin in postmortem brain tissue was significantly reduced by 38% in the hippocampus in early Alzheimer’s disease and 52% in the entorhinal cortex of definite Alzheimer’s disease compared to controls. It was observed that reduced levels of synaptotagmin were correlated with low Mini-Mental State Examination scores [301,302]. In a recent study, synaptotagmin 2 was significantly reduced in postmortem brain tissue of Alzheimer’s disease patients and reliably discriminated Alzheimer’s disease from Parkinson’s disease dementia [314]. Other studies have found similar observations in postmortem brain tissue, such as a 35% reduction in the cortex of patients with severe Alzheimer’s disease [269] and progressive decrement in patients with early Alzheimer’s disease with clinical dementia ratings of >1 [315].

Ohrfelt et al. assessed CSF synaptotagmin-1 levels and found a significant elevation in patients with both mild cognitive impairment and dementia due to Alzheimer’s disease. It was also observed that CSF synaptotagmin-1 levels in patients with dementia due to Alzheimer’s disease were significantly lower compared to those with mild cognitive impairment due to Alzheimer’s disease [316]. Interestingly, in a recent study, CSF synaptotagmin-1 levels were higher in Alzheimer’s disease patients compared to those with frontotemporal dementia, and there was a tendency to increased levels in patients with likely tau pathology [306].

In summary, studies have shown that synaptotagmin is reduced in the postmortem brain tissue of Alzheimer’s disease patients and may show a relationship with cognitive decline. CSF synaptotagmin is likely to be a promising biomarker as it is elevated in early-onset Alzheimer’s disease and there is an association with tau pathology.

### 7.4. Cerebrospinal Fluid Growth-Associated Protein 43 (GAP-43)

Growth-associated protein 43 (GAP-43), also identified as neuromodulin, is a 43 kDa neuron-specific presynaptic phosphoprotein found at high levels in neuronal growth cones and axon terminals in the adult human brain [317]. It is chiefly expressed in regions of the developed central nervous system that display high plasticity, such as olfactory bulbs, neocortex, entorhinal cortex, cerebellum, and hippocampus [318]. GAP-43 is involved in the regulation of the growth and development of neurons via the activity of protein kinase C, synaptogenesis, and nerve terminal plasticity, as well as memory and learning [319].

There are studies that have investigated the quantity and distribution of GAP-43 in postmortem brain tissue. Davidsson and Blennow reported a reduction of GAP-43 in the frontal cortex of early-onset and late-onset Alzheimer’s disease patients [255]. In a later study by the same authors, there was significantly decreased GAP-43 expression in the hippocampus (81% of control value) and frontal cortex (70% of control value), a positive correlation with duration of dementia, and a negative relationship with the number of senile plaques in the hippocampus [320]. Remarkably, in the investigation of neuroplasticity activity in the brain of Alzheimer’s disease, Rekart et al. found subfield-specific elevation of GAP-43 expression in the hippocampus and stratum lacunosum that was associated with the severity of the disease [321].

The level of CSF GAP-43 has been demonstrated to be elevated in Alzheimer’s disease and may be valuable in early disease detection [322,323,324]. Sandelius et al. recently reported significantly elevated levels of GAP-43 in Alzheimer’s disease compared to healthy controls, with patients with normal cognition and other neurodegenerative diseases. Higher GAP-43 levels were associated with the amount of Aβ plaques and neurofibrillary tangles but not with TDP-43 expression or CSF α-synuclein levels [322] (Table 3). There are other studies that have supported such observations. In an earlier study, CSF GAP-43 was significantly elevated in Alzheimer’s disease patients compared to age-matched healthy controls and patients with frontotemporal dementia. There was a highly significant association between GAP-43, CSF t-tau, and CSF soluble amyloid precursor protein in patients with neurodegenerative disorders, including vascular dementia [323] (Table 3). Likewise, in an explorative study involving 441 human samples of lumbar CSF taken antemortem and ventricular samples collected postmortem, CSF GAP-43 was elevated in two cohorts of preclinical and clinical Alzheimer’s disease matched to healthy controls. It was noted that there were significant elevations in CSF GAP-43 in dementia with Lewy bodies and Parkinson’s disease compared to controls [324]. Finally, in a recently published retrospective study, CSF GAP-43 was significantly elevated in mild cognitive-impaired Alzheimer disease patients and Alzheimer’s disease dementia patients compared to neurological controls and displayed good discriminatory power in making the distinction between patients with the disease and those with other dementias [325].

In summary, immunohistochemistry and quantitative tests on postmortem brain tissue found reduced GAP-43 in the hippocampus and frontal cortex, which was associated with senile plaques and duration of dementia. CSF GAP-43 levels are increased in Alzheimer’s disease patients compared with other neurodegenerative disorders such as Parkinson’s disease and frontotemporal dementia and may be useful in the differential diagnosis of the disease. Thus, the determination of CSF GAP-43 could possibly be of great significance in prospective tests for Alzheimer’s disease.

## 8. Neuronal Injury in Alzheimer’s Disease

### 8.1. Cerebrospinal Fluid Visinin-Like Protein 1 (VILIP-1)

Neuronal calcium sensor (NCS) proteins regulate Ca^2+^ in neurons of the central nervous system [270]. There are 14 neuronal calcium sensor proteins, divided into five subfamilies, namely, visinin-like protein 1 (VILIP-1), visinin-like protein 2 (VILIP-2), visinin-like protein 3 (VILIP-3), hippocalcin, and neurocalcin δ [271]. Visinin-like protein 1 is highly expressed in neurons within the central nervous system, and some of its physiological functions include the modulation of numerous cellular signal transduction pathways (including MAPK and cyclic nucleotide signaling involved in synaptic plasticity and the regulation of gene expression and monitoring membrane trafficking of ion channels and receptors as well as Ca^2+^ channels) [326].

The pathophysiology of Alzheimer’s disease entails disturbance in calcium homeostasis, with resulting alterations in neuronal Ca^2+^ [327]. Visinin-like protein 1 has been connected to mechanisms of a pathological nature, where neuronal Ca^2+^ homeostasis is negatively impacted, leading to neuronal injury and death [270]. Perturbations in calcium homeostasis in the central nervous system and the neurotoxic role of VILIP-1 have been implicated in neurodegenerative diseases such as Alzheimer’s disease [328]. Damage to neurons in the brain that contain VILIP-1 leads to increase levels of this biomarker in CSF, which can be measured using commercially available assays [329]. VILIP-1 may play a significant role in mediating calcium-dependent neurotoxicity and the pathological changes observed in patients with Alzheimer’s disease [271].

Visinin-like protein 1 recently emerged as a potential marker for Alzheimer’s disease [330]. In a study comprising 33 Alzheimer’s disease patients and 24 normal controls, CSF VILIP-1 levels were substantially higher in the Alzheimer’s disease patients compared with controls. The diagnostic performance of CSF VILIP-1 was comparable to that of core neurodegenerative biomarkers such as p-tau, t-tau, and Aβ_42_, and it showed a high degree of association with t-tau (r = 0.605) and p-tau (0.809) [331] (Table 3). In a later study that examined CSF VILIP-1 as a diagnostic and prognostic marker of Alzheimer’s disease, elevated CSF VILIP-1 levels in Alzheimer’s disease patients discriminated them from cognitively normal controls. There were correlations between CSF VILIP-1 levels and CSF t-tau, p-tau-181, and brain volume in patients with Alzheimer’s disease, and the VILIP-1/Aβ_42_ ratio was prognostic for future cognitive impairment in normal individuals [332]. There were significantly higher levels of CSF VILIP-1 levels in patients with Alzheimer’s disease compared with control [333] and approximately 50% higher levels and moderate association with Alzheimer’s disease patients compared with controls [27]. However, baseline CSF VILIP-1 levels were comparable between Alzheimer’s disease patients and cognitively normal individuals and predicted progression from mild cognitive impairment to Alzheimer’s disease [153].

Persons with mild cognitive impairment are at a higher risk of developing Alzheimer’s disease dementia due to the pathophysiological processes that are involved in neurodegeneration. There is a growing necessity to find CSF biomarkers that can identify these individuals for early intervention [272]. In a study by Mroczko et al., Alzheimer’s disease patients had significantly higher levels of CSF VILIP-1 than persons with mild cognitive impairment and normal cognitive function. The elevated VILIP-1 levels were significantly correlated with higher p-tau-181 and a reduced Aβ_42_/Aβ_40_ ratio in the Alzheimer’s disease patients, indicating its role in the pathophysiology of the disease [334]. Likewise, a recent study of the ADNI cohort found that CSF VILIP-1 levels at baseline were higher in Alzheimer’s disease patients and mild cognitive impaired individuals with amyloid-β positivity compared with cognitively normal (Aβ-negative) and amyloid-β-negative mild cognitive-impaired persons [190]. It is noted that there were no significant differences in CSF VILIP-1 levels among Alzheimer’s disease patients, mild cognitive-impaired individuals, and cognitively normal individuals who displayed amyloid-β positivity [190]. However, in another study, there was no significant difference in CSF VILIP-1 levels between mild cognitive impairment patients and those with Alzheimer’s disease [335].

There are corresponding clinical features between Alzheimer’s disease and other primary degenerative dementias that make differentiation challenging in the clinical environment [336]. Persons with mild cognitive impairment are at a higher risk of developing Alzheimer’s disease dementia due to the pathophysiological processes involved in neurodegeneration. There is a growing necessity to find CSF biomarkers that can identify these individuals for early intervention [272]. Investigating the diagnostic utility of biomarkers of neuronal injury such as VILIP-1 could expedite clinical diagnosis [337,338]. Using commercial ELISA kits to investigate the diagnostic accuracy of CSF VILIP-1, levels of the biomarker was significantly higher in Alzheimer’s disease patients compared to both dementia with Lewy bodies patients and normal controls [339] (Table 3). In addition, the CSF VILIP-1 levels were positively correlated with CSF p-tau-181 and t-tau proteins, and the CSF VILIP-1/Aβ_42_ ratio had reasonably good diagnostic accuracy to permit the identification and differential diagnosis of Alzheimer’s disease [339]. CSF VILIP-1 discriminated Alzheimer’s disease patients from those with dementia with Lewy bodies with a sensitivity of 77.1% and a specificity of 100% [335]. Other support comes from a study by Tarawneh et al. (2011) [332], where CSF VILIP-1 levels correlated with CSF p-tau-181 and t-tau proteins, which differentiated individuals with Alzheimer’s disease from other types of dementias. Remarkably, CSF VILIP-1/Aβ_42_ had similar predictive values as p-tau-181 and t-tau proteins for future cognitive impairment in normal individuals [332].

In a longitudinal study, there was a reduction in CSF VILIP-1 levels in symptomatic patients with late-onset Alzheimer’s disease but not in mild cognitive impairment or cognitively normal groups [190]. Moreover, CSF VILIP-1 may be predictive of future cognitive loss in individuals with normal cognitive function, similar to t-tau protein [332], and along with VILIP-1/Aβ_42_, may be prognostic of rates of global cognitive decline [340]. In another longitudinal observational study, baseline CSF VILIP-1 gave predicted rates of whole-brain and regional atrophy over a mean follow-up period of 2–3 years [341]; this was associated with progression from mild disease progression to Alzheimer’s disease [153].

### 8.2. Cerebrospinal Fluid Neurofilament Light

Neurofilaments are type IV intermediate filaments located in the cytosol of neurons and are plentiful in axons. They are heteropolymers that are comprised of four polypeptide subunits (specifically, light (68 kD), middle (95 kD), and heavy (115 kD) neurofilaments) and α-internexin in the central nervous system [342]. In the peripheral nervous system, in addition to heavy, middle, and light neurofilaments, there is peripherin [343]. Neurofilaments possess elastic fibrous properties and long half-lives that allow them to maintain the shape of neurons. In addition, they regulate axonal caliber, control transmission of electrical impulses along axons and synaptic plasticity, determine the movement of synaptic vesicles, and contribute overall to regular synaptic function [344,345]. Progressive alterations of neurofilaments, particularly in human hippocampal neurons and their aggregation, subsequently cause neuronal degeneration and the formation of neurofibrillary tangles in neurodegenerative diseases such as Alzheimer’s disease [273,343]. Extensive disruption in neurofilament transport and metabolism in the central and peripheral nervous systems is observed in patients with Parkinson’s disease as altered neurofilament-rich Lewy bodies are located in the pigmented subcortical neurons of these patients [346].

Pijnenburg et al. investigated the discriminative ability of CSF neurofilaments in patients with early-onset Alzheimer’s disease and healthy control individuals with normal cognitive function. Cerebrospinal fluid neurofilament light levels were significantly greater in patients with early-onset Alzheimer’s disease compared to cognitively healthy controls [347]. In a recent study by Dhiman et al. that examined the clinical utility of CSF neurofilament light in the diagnosis of Alzheimer’s disease, the levels of this biomarker and the neurofilament light/Aβ_42_ ratio were significantly greater in patients with the disease compared to cognitively healthy controls. It was noted that both CSF neurofilament light levels and neurofilament light/Aβ_42_ ratios predicted cognition, cortical amyloid load, and brain atrophy [348]. In a multicenter cross-sectional study, CSF neurofilament light levels were also discriminative of patients with Alzheimer’s disease that were stratified by CSF t-tau protein and CSF Aβ_42_ levels compared to cognitively healthy control persons [349] (Table 3). The meta-analysis by Olsson et al., involving 13,018 healthy controls and 15,699 Alzheimer’s disease patients, established that CSF neurofilament light levels had a large effect size, discriminating between the two groups [27]. Moreover, the neurofilament heavy isoform in CSF was elevated in Alzheimer’s disease patients compared to healthy controls [350] and reference cohorts [351].

The neurodegenerative process in Alzheimer’s disease happens early in development. Detecting deviations in CSF biomarkers such as neurofilament light, a marker of neuroaxonal degeneration, may enhance the prediction of evolution from mild cognitive impairment to dementia [352]. Zetterberg et al. investigated whether CSF neurofilament light levels are related to a decline in cognition in a cohort of patients with Alzheimer’s disease, mild cognitive impairment, and healthy individuals with normal cognitive function. CSF neurofilament light levels were greater in the Alzheimer’s disease dementia, stable, and progressive mild cognitive impairment groups compared to the control group [353]. It is noteworthy that the elevated CSF neurofilament light level was concomitant with faster disease progression, white matter intensity change, and brain atrophy over time [353]. In a recent case–control study with a follow-up of 1 to 18 years, there was a stepwise increase in CSF neurofilament light levels between control individuals, participants with mild cognitive impairment, and those with Alzheimer’s disease. This suggests that CSF neurofilament light levels give an indication of the strength of the neurodegenerative processes in Alzheimer’s disease [354]. Other studies found that CSF neurofilament light levels differ in all stages within the Alzheimer’s disease continuum [355], and patients with converting mild cognitive impairment and stable dementia had higher CSF neurofilament light levels compared to controls and participants with nonprogressive mild cognitive impairment [274]. However, in a longitudinal study that assessed changes in CSF neurofilament level, the biomarker could not differentiate between patients in the control, Alzheimer’s disease, and mild cognitive impairment groups [356].

Loss of cortical neurons is a key pathological feature in neurodegenerative dementias. CSF neurofilaments are a biomarker of axonal loss and death of neurons, and their clinical utility has been assessed in cohorts of patients with different dementias [194,357]. Alcolea et al. found high CSF neurofilament light levels in patients with frontotemporal dementia and Alzheimer’s disease compared to controls. The CSF neurofilament light/soluble β fragment of the amyloid precursor protein ratio distinguished patients with frontotemporal dementia from those with Alzheimer’s disease [194]. In an earlier study, CSF neurofilament light levels were highest in patients with frontotemporal dementia, vascular dementia, mixed vascular dementia, and Alzheimer’s disease. Elevated CSF neurofilament light levels were associated with low cognition, short survival time, and disease severity, particularly in patients with Alzheimer’s disease [358]. Similarly, CSF neurofilament heavy levels were elevated in patients with Alzheimer’s disease and vascular dementia compared to control [350].

There are meta-analyses of studies and systematic reviews that have shown higher levels of CSF neurofilament light and heavy levels in Alzheimer’s disease, frontotemporal lobe dementia, and vascular dementia [359] and highest levels of CSF neurofilament light in Huntington’s disease, amyotrophic lateral sclerosis, and frontotemporal dementia compared to controls [360]. In both meta-analyses, CSF neurofilament light levels distinguished patients with frontotemporal lobe dementia from those with Alzheimer’s disease [359,360]. However, in a recent systematic review and meta-analysis, Forgrave et al. found that CSF neurofilament levels provided no discriminatory utility for Alzheimer’s disease [361]. Furthermore, CSF neurofilament light levels were not able to differentiate Alzheimer’s disease from dementia with Lewy bodies and progressive nonfluent aphasia [362].

In summary, CSF neurofilament light is elevated in patients with Alzheimer’s disease and provides an indication of axonal degeneration and neuronal death. The biomarker is predictive of the progression from mild cognitive impairment to dementia and is associated with short survival support and disease severity. Therefore, it could be used to monitor disease progression in Alzheimer’s disease. However, the diagnostic potential of CSF neurofilament light is not superior to other established CSF markers and lacks adequate specificity in differentiating Alzheimer’s disease from other neurodegenerative dementias, including frontotemporal dementia and amyotrophic lateral sclerosis.

## 9. α-Synuclein Pathology in Alzheimer’s Disease

α-Synuclein is a natively unfolded neuronal protein consisting of 140 amino acid residues, encoded by the SNCA gene, and its function involves synaptic vesicle fusion and the release of neurotransmitters [363]. It is located in the presynaptic terminals of neurons and is expressed throughout the brain, with high levels in the hippocampus, thalamus, neocortex, and substantia nigra but low levels in glial cells [364]. In the cytosol of neurons, its primary unfolded monomer form can undergo a conformational transformation in the presence of lipid membranes into a folded α-helical secondary structure that is disposed to developing dimers and oligomers [365]. Pathological processes comprising α-synuclein aggregation and toxicity have been implicated in the development of a number of neurodegenerative diseases. Post-translational modifications of α-synuclein include phosphorylation (mainly at serine residues S129), ubiquitination (where ubiquitin is attached to α-synuclein at lysine residues (K6, K10, and K12)), and nitration (where a nitro molecule is attached to α-synuclein at tyrosine residues (Y136, Y133, Y125, and Y39)) [366,367,368]. These post-translation modifications result in the aggregation of α-synuclein, changes in its function, activity, and degradation processes, and an increase in neurotoxic species, apoptosis, and cell death. Interestingly, the nitration of α-synuclein, particularly in an environment of oxidative stress, is widely recognized as a significant feature in Lewy body diseases [369].

There is increasing evidence that α-synuclein, performing as a prion-like pathological agent and by its aggregation and misfolding in the peripheral nervous system due to genetic and environmental factors, causes the degeneration of neurons in the substantia nigra of the brain. The ongoing α-synuclein pathology results in worsening brain functions and cognition [370,371]. Furthermore, the chief constituents found in Lewy bodies are nitrated aggregates of α-synuclein, which are characteristics of a group of neurodegenerative diseases commonly known as α-synucleinopathies. Primary α-synucleinopathies comprise dementia with Lewy bodies, Parkinson’s disease, multiple system atrophy, and Parkinson’s disease dementia [372]. In some other neurodegenerative disorders, such as Alzheimer’s disease, α-synuclein aggregates are present, and these are termed secondary α-synucleinopathies [373].

### Cerebrospinal Flzuid α-Synuclein

There are several investigators that have examined α-synuclein levels in the brain and CSF of patients with Alzheimer’s disease and primary α-synucleinopathies such as Parkinson’s disease and dementia with Lewy bodies [374,375,376]. Majbour et al. determined CSF total α-synuclein in a cohort of Alzheimer’s disease patients and cognitive-intact controls. CSF total α-synuclein levels were significantly higher in Alzheimer’s disease patients compared to controls, with a sensitivity of 85% and a specificity of 84% (AUC = 0.88). The CSF total α-synuclein levels were positively correlated with t-tau and p-tau-181 proteins and negatively correlated with baseline Mini-Mental State Exam scores in patients with Alzheimer’s disease [374]. These observations were supported by another study, where CSF α-synuclein levels were greater in Alzheimer’s disease patients compared to healthy controls with normal cognitive function. Furthermore, CSF α-synuclein levels were significantly correlated with t-tau and p-tau-181 protein levels in autopsy-confirmed Alzheimer’s disease patients [375] (Table 3). However, in a review and meta-analysis study, there were no differences in CSF α-synuclein levels in Alzheimer’s disease patients and controls with normal cognitive function [376]. Surprisingly, in an earlier study, CSF α-synuclein levels in Alzheimer’s disease patients were significantly lower compared to controls [377].

Individuals with mild cognitive impairment present with short-term memory loss and are at risk of developing Alzheimer’s disease or other neurodegenerative disorders [378]. In a study by Korff et al., CSF α-synuclein levels were significantly higher in individuals with mild cognitive impairments and Alzheimer’s disease patients compared to controls [379] (Table 3). CSF α-synuclein levels had a sensitivity of 65% and a specificity of 74% as a diagnostic marker of Alzheimer’s disease and were negatively correlated with Mini-Mental State Exam scores [379]. In a cross-sectional study involving patients from the Dominantly Inherited Alzheimer’s Network, sporadic mild cognitive-impaired patients had higher baseline CSF α-synuclein levels, which were inversely associated with their Mini-Mental State Examination scores, compared with controls with normal cognitive functions [380]. Notably, the CSF α-synuclein levels were associated with t-tau proteins and Aβ_40_ levels. This indicates the contribution of the biomarker to the onset of cognitive symptoms and the pathophysiology of the initial stages of Alzheimer’s disease [380].

There is supporting evidence that CSF α-synuclein enhances the prognostic and diagnostic performance of CSF t-tau proteins and amyloid-β peptides in Alzheimer’s disease. In the ADNI cohort study, CSF α-synuclein in Alzheimer’s disease patients and individuals with mild cognitive impairment was positively correlated with p-tau-181 protein [381]. Moreover, a longitudinal follow-up for 7 years of patients in the ADNI cohort found that CSF α-synuclein predicted a decline in cognitive function and progression from mild cognitive impairment [382]. On the other hand, a longitudinal study conducted by Toledo et al. found no significant differences in mean CSF α-synuclein levels at baseline between controls with normal cognitive functions, patients with mild cognitive impairment, and Alzheimer’s disease patients [381].

The determination of biomarkers in CSF, such as α-synuclein, is potentially valuable for the early diagnosis of patients with primary and secondary α-synucleinopathies [383]. Kasuga et al. investigated the discriminating utility of CSF α-synuclein in patients with primary α-synucleinopathies such as dementia with Lewy bodies and secondary α-synucleinopathies, including Alzheimer’s disease. CSF α-synuclein levels in patients with dementia with Lewy bodies were significantly lower than those in patients with Alzheimer’s disease and other dementias [384]. Similarly, in an earlier cross-sectional study, mean CSF α-synuclein values were lower in patients with Parkinson’s disease and dementia with Lewy bodies compared to Alzheimer’s disease patients or non-neurodegenerative disease controls [385]. Moreover, there are other studies that have found similar observation of higher CSF α-synuclein levels in Alzheimer’s patients compared to patients with primary α-synucleinopathies such as Parkinson’s disease, multiple system atrophy, and dementia with Lewy bodies [275,375,376,377]. However, there are studies that have reported discordant findings. Kapaki et al. found significantly higher CSF α-synuclein levels in patients with dementia with Lewy bodies compared to Alzheimer’s disease patients. The elevated CSF α-synuclein levels were 90% sensitive and 50% specific in the discrimination of dementia with Lewy bodies versus Alzheimer’s disease [386]. Other studies have found that CSF α-synuclein levels did not differ significantly between dementia with Lewy bodies and Alzheimer’s disease patients [387] and between Alzheimer’s disease patients and patients with Parkinson’s disease and dementia with Lewy bodies [388].

In summary, CSF α-synuclein may be potentially useful for the diagnosis of Alzheimer’s disease as its levels are increased in patients with the disorder. CSF α-synuclein levels were significantly greater in Alzheimer’s disease compared with primary α-synucleinopathies such as dementia with Lewy bodies, Parkinson’s disease, and multiple system atrophy. The associations of CSF α-synuclein with established biomarkers such as t-tau protein and Aβ_40_ could somewhat improve the differential diagnosis of Alzheimer’s disease with the primary α-synucleinopathies and better predict longitudinal changes in cognition.

## 10. Transactive Response DNA Binding Protein 43 Pathology

Transactive response DNA binding protein (TDP-43) in humans is encoded by the TARDBP gene and comprises 1414 amino acid residues in 4 domains and a molecular weight of 44.74 kDa [276]. TDP-43 exerts its biological effect via its binding of both single-stranded RNA and DNA and proteins. It has been associated with the regulation of gene transcription, modulating gene splicing and maintaining mRNA stability, as well as transport and local translation in neurons [389]. Abnormal post-translational modifications, comprising hyperphosphorylation, ubiquitination, and abnormal cleavage and/or nuclear reduction of TDP-43 in glial cells and neurons, are recognized as the characteristics for TDP-43 inclusions [390].

Over the last ten years, there is increasing evidence that TDP-43 proteinopathy is involved in the pathogenesis of a number of neurological disorders, with subsequent TDP-43 nuclear depletion, accumulation of insoluble aggregates in cytoplasm, and neurodegeneration [391]. Ubiqitin inclusions of TDP-43 have been found in patients with amyotrophic lateral sclerosis and frontotemporal lobar degeneration [392,393]. Joseph et al. detected TDP-43-positive inclusions in patients with Alzheimer’s disease and, in developing a staging scheme comprising of TDP-43, noted its deposition in a stereotypic fashion over five distinct topographic stages [394]. It is worth remarking that data from the Rush Memory and Aging Project and Religious Orders Study showed that cytoplasmic hyper-phosphorylated TDP-43 inclusions were associated with a higher likelihood of clinical expression of Alzheimer’s-type dementia [395].

### Cerebrospinal Fluid Transactive Response DNA Binding Protein 43 (TDP-43)

A number of studies have determined the frequency of Alzheimer’s disease with TDP-43 inclusions using immunohistochemistry [395,396,397]. TDP-43 pathology has been identified in 23–52% of Alzheimer’s disease patients [394,395,396,397] and appears to be concomitant with decreased cognitive function, increased brain atrophy, and memory loss [394]. McAleese et al. investigated TDP-43 pathology prevalence and severity in a number of patients with various neurological disorders by examining 119 human postmortem brains. The prevalence of TDP-43 pathology was significantly greater in Alzheimer’s disease patients compared with similar-age controls with normal cognitive function [398]. In addition, the prevalence of TDP-43 pathology was significantly higher in patients with mixed dementia with Lewy bodies and Alzheimer’s disease, and advanced age at death was associated with higher TDP-43 pathology in Alzheimer’s disease patients [398]. Similarly, in an earlier study, insoluble TDP-43 was found in the parietal neocortex of subjects with mild cognitive impairment and Alzheimer’s disease and was positively correlated with amyloid plaques, soluble Aβ42, and tau filaments. The frequencies of persons with phosphorylated-TDP-43 or TDP-43 were greater in Alzheimer’s disease patients than in controls with no cognitive impairment [399]. These observations have been corroborated by Chen-Plotkin et al.; they have stated that TDP-43 pathology accompanies neurofibrillary tangles and amyloid plaques in Alzheimer’s disease [400].

In summary, these studies provide evidence of the involvement of TDP-43 proteinopathy in the development of Alzheimer’s disease. TDP-43 inclusions have been identified in postmortem brains, and TDP-43 pathology may be applied to Alzheimer’s disease staging. The prevalence of TDP-43 pathology was significantly greater in Alzheimer’s disease patients and may be associated with amyloid and tau pathology. Research with serum and CSF TDP-43 may improve its value as a biomarker for the diagnosis and prognosis of Alzheimer’s disease.

## 11. CSF Biomarkers Associated with Iron Metabolism

### 11.1. Iron Toxicity and Alzheimer’s Disease

There is extensive evidence from a large number of studies that an abnormality of iron metabolism in the brain gives rise to oxidative stress due to reactions catalyzed by iron in elevated amounts, impairment in neuronal function, and, subsequently, neuronal cell death [401]. Imbalance of iron homeostasis in the brain can initiate the synthesis and accumulation of Aβ plaques, promote the formation of neurofibrillary tangles due to the phosphorylation and accumulation of tau proteins [402,403], and stimulate ferroptosis (where severe lipid peroxidation occurs with the associated free radical involvement) [404]. These factors are key in the pathogenesis of Alzheimer’s disease and other chronic neurologic disorders [405]. Moreover, neuroferritinopathy is a degenerative disorder in adults, with an etiology of elevated ferritin, associated with iron release, that is linked to lipid peroxidation, cell damage due to reactive oxygen species generation, and elevated iron-containing lipoxygenase that culminates in programmed neuronal death [406].

Neuroferritinopathy has been implicated in the pathology of several neurodegenerative disorders such as Parkinson’s disease and Alzheimer’s disease and is concomitant with behavioral aberrations, cognitive impairment, and motor dysfunction [407].

### 11.2. Cerebrospinal Fluid Iron Ferritin

The dysregulation of iron homeostasis is one of the risk factors in the pathology of Alzheimer’s disease as this transition metal stimulates the misfolding of β-amyloid and the formation of plaque aggregates [408]. Ayton et al. examined the association between CSF and longitudinal alterations in CSF t-tau proteins and β-amyloid in 296 subjects with baseline values over a period of up to 5 years [409]. Elevated CSF ferritin in subjects with Alzheimer’s disease pathology was significantly positively associated with elevated Aβ_42_ plaque formation but not with longitudinal variations in CSF Aβ_42_ or t-tau in those individuals with low baseline pathology [409] (Table 3). In an earlier study, Ayton et al. investigated whether ion status in the brain influences longitudinal disease progression and outcomes in subjects in the ADNI cohort. Baseline levels of CSF ferritin were associated with a decline in cognition and projected disease progression from mild cognitive impairment to Alzheimer’s disease [410]. Interestingly, there were no differences in CSF ferritin levels among Alzheimer’s disease patients and subjects with mild cognitive impairment compared to controls. Notably, CSF ferritin was greater in individuals who were carriers of the APOE ε4 gene compared with noncarriers [410]. In a later report by the same research group, subjects with the APOE ε4 allele, with an augmented risk of Alzheimer’s disease, had elevated levels of CSF ferritin and cognitive decline [411]. However, CSF ferritin was not increased in a large clinical cohort of Alzheimer’s disease patients [412].

There are studies that have reported variable elevations of iron in numerous cortical regions of the brains of Alzheimer’s disease patients postmortem [413]. Iron was significantly elevated in the frontal cortex and globus pallidus of postmortem brains of Alzheimer’s disease and the globus pallidus of patients with Parkinson’s disease [414]. There are studies that have reported elevated iron levels in the brains of Alzheimer’s disease patients [415,416] and elderly subjects with mild cognitive impairment compared with controls [417]. In the study by van Bergen et al., cognitive-impaired subjects with the apolipoprotein E ε4 (APOE-e4) allele presented with higher iron and amyloid-β plaque-loads in the subcortical and cortical regions of the brain and greater risk for neurocognitive dysfunction owing to Alzheimer’s disease [417]. Likewise, the concentration of ferritin light chains was significantly correlated with neuronal loss and the quantity of senile Aβ amyloid plaques in the CA1, CA2, and subiculum sectors of the hippocampus [418]. In addition, a diversity of imaging techniques, such as magnetic resonance imaging and phase imaging, has been used to quantitatively determine iron accumulation in different regions of the brain, such as hippocampus, pulvinar thalamus, pulvinar, and temporal cortex [278,419,420,421]. The concentrations of brain iron were positive associated with the severity of impairment in cognition [278,421] and negatively associated with the extent of the disease [420].

Abnormal regulation of iron in different regions of the brain may be involved in the pathogenesis of neurodegenerative disorders such as Alzheimer’s disease and Parkinson’s disease [414]. Using subjects in the ADNI study, Diouf et al. longitudinally examined whether levels of CSF ferritin were related to the progression of the prodromal disease of individuals with extraordinary β-amyloid pathology using recognized cut-off values of the CSF t-tau/Aβ_42_ ratio [422]. Baseline CSF ferritin was concomitant with hypometabolism (measured using PET scans of fluorodeoxyglucose) in individuals with high t-tau/Aβ_42_ [422]. The authors suggested that elevated neuronal iron may have a critical role in the physiopathology of Alzheimer’s disease [422]. Moreover, there was a significant elevation in CSF ferritin in Alzheimer’s disease patients compared with patients with Parkinson’s disease and controls [423] (Table 3).

In summary, the findings of these studies present that iron is involved in the pathophysiology and neuropsychological dysfunction of Alzheimer’s disease. CSF ferritin levels may be a beneficial biomarker of Alzheimer’s disease as it is predictive of progression from mild cognitive impairment to Alzheimer’s disease and other outcomes of Alzheimer’s disease. CSF ferritin is associated with amyloid-β and tau pathology and may be useful in detecting the early stages of Alzheimer’s disease.

## 12. Emerging Biomarkers

### Cerebrospinal Spinal MicroRNAs

MicroRNAs (miRNAs) are minute noncoding RNAs of 20–22 nucleotides that bind their target mRNAs and modulate posttranscriptional gene expression via the establishment of an RNA-induced silencing complex [424]. miRNAs are stable in CSF as they are present in ribonucleoprotein complexes or exosomes secreted by glia and neurons, and they are detected in low concentrations using quantitative PCR [425]. A number of studies have shown alterations in the expression of various miRNAs and have suggested a role in the pathogenesis of a number of neurodegenerative disorders, including Alzheimer’s disease [426]. Zhang et al. conducted a systematic review and meta-analysis of 10 studies involving 770 Alzheimer’s disease patients and 664 normal controls and demonstrated that miRNAs presented remarkable diagnostic performance, with a specificity of 80%, specificity 83%, and a diagnostic odds ratio of 14 (95% CI: 11–19) [427].

Lusardi et al. used TaqMan^®^ arrays to profile miRNAs in 50 Alzheimer’s disease patients and 49 controls and identified 36 CSF miRNAs that discriminate the disease from controls [428] (Table 3). The researchers found that the combination of miRNAs improved their sensitivity and specificity and the addition of the ApoE genotype increased the classification [428]. In an earlier study, quantitative reverse transcriptase-polymerase chain reaction (qRT-PCR) identified significantly lower levels of miR-146a, miR-34a, and miR-125b, as well as higher levels of miR-29b and miR-29a, compared to controls [429]. Similarly, in an explorative pilot study using real-time quantitative polymerase chain reactions, there was significant upregulation of miR-15a-5p and let-7i-5p and significant downregulation of miR-29c-3p in Alzheimer’s disease patients compared to controls [430] (Table 3). Other miRNAs, such as CSF miRNA-125b, miRNA-155, miRNA-9, and miRNA-146a, measured using fluorescent miRNA-array-based analysis, were upregulated in Alzheimer’s disease patients compared to controls and were associated with neuroinflammation and neurodegeneration [431]. Furthermore, studies have reported decreased expressions of CSF hsa-miR-27a-3p [432], miR-26b and miR-125b [433], miR-384 [277], miRNA-9, and miR-101 [434], as well as upregulation of miR-206 [435], miR-34c [434], miR-125b, and miR-222 [436] in Alzheimer’s disease patients compared to controls.

Open array technology has been employed in microRNA profiling of CSF, and Denk et al. identified 1178 unique miRNAs of which miR-1274a, miR-100, and miR-146a were differentially expressed in Alzheimer’s disease patients [437]. The miRNAs that were identified as informative included miR-103, miR-219, miR-219, miR-275, miR-296, miR-375, miR-505, miR-708, miR-766, miR-4467, and miR-3622b-3p. There was significant upregulation of CSF miR-146a that was inversely associated with tau and Aβ_42_, and a combination of CSF miR-375, miR-100, and miR-103 identified Alzheimer’s disease cases with an accuracy of 95.5% [437]. In a recent study, McKeever et al. examined miRNA expression profiles in CSF-derived exosomes from young-onset Alzheimer’s disease (YOAD) and late-onset (LOAD) patients [279]. There were decreased expressions of miR-605-5p, miR-16-5p, and miR-451a and upregulation of miR-125b-5p in YOAD patients compared to controls. In the cohort patients with LOAD, there was increased expression of miR-125b-5p and downregulation of miR-605-5p and miR-451a compared to controls [279]. The authors suggested that these miRNAs signify novel targets for unraveling the mechanism of Alzheimer’s disease and the development of potential biomarkers, particularly exosomal miR-16-5p, which is related to YOAD [279].

## 13. Future Perspective

Significant research is currently being pursued in the area of Alzheimer’s disease biomarkers; future directions involve the discovery of biomarkers that can evaluate the entire continuum of disease pathogenesis and enable accurate diagnosis in the early stages of the condition. Laboratory assays for blood-based Alzheimer’s disease biomarkers are presently being developed, and they may be of assistance in monitoring asymptomatic persons and diagnosing those who are symptomatic. The challenges of lower concentrations of Alzheimer’s disease biomarkers in the plasma compared with CSF, a more complex matrix, as well as analytical and biological variabilities need to be addressed. Clinical-grade assays on automatic platforms used in the measurement of plasma biomarkers need to be properly validated. There is recently published literature on the use of immune-magnetic reduction assays and single-molecule array technology for plasma-based Alzheimer’s disease biomarker measurement [438]. Furthermore, modern advances in the development of proteomics, metabolomics, mass spectrometry, and the use of exosomes have shown significant possibilities for blood-based biomarkers as screening tools for Alzheimer’s disease.

Beyond the core CSF biomarkers, there are other analytes associated with amyloidogenesis and/or Aβ metabolism, neuroinflammation, and pathological oxidative changes that have shown potential and are worth future investigation. Aβ oligomers in blood and CSF have been determined by the use of single-molecule fluorescence microscopy and enzyme-linked immunosorbent assays (ELISAs). There are many methodological challenges, and, recently, there have been reports on improved methods for measuring Aβ oligomers and pathological misfolded tau. The protein misfolding cyclic amplification assay is extremely sensitive in identifying Aβ oligomers in the CSF of Alzheimer’s disease patients [280]. In the last few years, a real-time quaking-induced conversion assay has been employed in the ultrasensitive detection of tau aggregates that provides molecular evidence of tau filament propagation in the development of Alzheimer’s disease [439]. Although these molecular protocols are ultrasensitive and improve the detection of CSF biomarkers, there is a need for method validation and measurement of both Aβ oligomers and tau aggregates in clinical studies with large populations of Alzheimer’s disease patients. Future longitudinal studies are warranted as they are critical for evaluating the association between the detection of these biomarkers and disease progression.

The application of metabolomics offers a novel approach to detecting metabolites such as amino acids in CSF and plasma of Alzheimer’s disease patients. There are studies that have found increased amino acid levels in Alzheimer’s disease patients compared to individuals with mild cognitive impairment [440] and controls [441]. These metabolites correlate with disease severity. Metabolomics in combination with CSF core biomarkers and advanced imaging techniques could improve diagnostic accuracy and prognosis, as well as monitor the effectiveness of treatment intervention [442]. Likewise, an evolving area of Alzheimer’s disease research is the use of integrative proteomics, specifically multiplex mass spectrometry, to identify CSF biomarkers that span the wide continuum of Alzheimer’s disease pathophysiology [443]. There is the development of metabolic and synaptic panels of protein biomarkers that reflect specific pathophysiological processes [444]. These findings are very promising and point to the possible development of a network-based protein biomarker tool that could be applied to the diagnosis and prognosis of Alzheimer’s disease in research and also in clinical settings.

The standardization of clinically based assays and methodologies for new Alzheimer’s disease biomarkers is very important, and the tests must be validated with defined sensitivity and specificity. The validation of these analytes should be conducted in well-designed clinical trials, with large cohorts and close attention given to covariates such as gender, ethnicity, age, and APOE genotype. Longitudinal studies investigating different Alzheimer’ disease biomarkers in body fluids may result in the development of diagnostic and prospective panels for the staging of the disease, particularly in the very early and preclinical phases. The implementation of validated biomarkers in different body fluids, with high sensitivity and specificity for Alzheimer’s disease in the primary care setting that results in improved diagnosis, prognosis, and monitoring of therapeutic intervention, is one of the main goals of this area of research.

## 14. Conclusions

In conclusion, a large number of miRNAs have been identified using methods such as real-time PCR, and their altered expressions have been observed in the early and late stages of Alzheimer’s disease, suggesting their participation in the pathogenesis of this neurodegenerative disorder. The miRNAs in total CSF and those in CSF-derived exosomes are novel and potential diagnostic markers for Alzheimer’s disease, and their combination increases the sensitivity and specificity of their performance. However, there is a need for employing more effective molecular methods that improve detection and performing more confirmation studies using large cohorts of Alzheimer’s disease patients.

The complex pathophysiology of Alzheimer’s disease is reflected by alterations in the composition of CSF due to the formation and deposition of amyloid plaques, synthesis of neurofibrillary tangles, neuronal injury and synaptic loss, disturbance in lipid metabolism, neuroinflammation, and gliosis. Currently, Aβ_42_, t-tau, and p-tau are the classical and core biomarkers for the diagnosis of Alzheimer’s disease. Findings of numerous cross-sectional investigations suggest a model of ongoing reductions in CSF Aβ_42_ due to the formation of amyloid plaques in the initial and preclinical stages. In the symptomatic phase, CSF Aβ_42_ levels remain fairly constant, with accompanied elevations of t-tau and p-tau, which are markers of neuronal injury. Notably, CSF Aβ_42_, t-tau, and p-tau have been included in the diagnostic criteria for Alzheimer’s disease. Along with amyloid positron emission tomography scans, they are presently used in the clinical diagnosis of symptomatic Alzheimer’s disease patients. The combination of these CSF biomarkers has resulted in improved sensitivity and specificity than when used alone and is able to predict the progression from mild cognitive impairment to Alzheimer’s disease.

Over the past ten years, several novel biomarkers of Alzheimer’s disease pathogenesis have been identified that can provide greater accuracy in diagnosis and prognosis in study cohorts and a better understanding of the neuropathologic changes during the development of the condition. The investigation of these novel CSF biomarkers and their association with the classical Alzheimer’s disease triad could signify further development in their clinical applications. Novel CSF biomarkers are necessary in order to advance the differential diagnosis and prognosis of Alzheimer’s disease with a satisfactory discriminating prospect for this condition compared with other neurodegenerative diseases. Levels of CSF hFABP and YKL-40 are suitable markers for the diagnosis of Alzheimer’s disease and are able to discriminate it from other neurological conditions. Increased CSF YKL-40 predicts the progression from mild cognitive impairment to clinical Alzheimer’s disease and has been concomitant with humoral immunity.

The association of novel biomarkers such as CSF MCP-1 and neurogranin with core biomarkers such as t-tau and p-tau has improved their diagnostic accuracy for discriminating between Alzheimer’s disease and dementia with Lewy bodies. CSF MCP-1 and neurogranin are elevated in the early and late stages of Alzheimer’s disease and are associated with an enhanced rate of cognitive decline and neurodegeneration. They complement CSF Aβ_42_, p-tau, and t-tau protein levels, which make them possibly valuable biomarkers for monitoring the progression of the condition. Notably, they may be valuable additions as auxiliary tests to the current panel of core biomarkers; however, further validation is required in large clinical trials.

Although CSF progranulin may be elevated during the progress of Alzheimer’s disease and may add to its risk, further investigations are warranted to examine its potential for assessing disease severity and early detection. Likewise, CSF VILIP-1 is associated with p-tau and t-tau, which supports the principle that it is a biomarker of neuronal injury. Studies have shown that it is increased in Alzheimer’s disease patients compared with controls and is useful in discriminating Alzheimer’s disease from other dementias. More longitudinal studies are needed to ascertain its ability to predict future cognitive loss in persons with normal cognitive function who develop the disease.

Elevated CSF osteopontin and GAF levels are established in Alzheimer’s disease patients and subjects with mild cognitive impairment who subsequently develop the condition. The former test is a biomarker of the prodromal Alzheimer’s disease stage, and they are both beneficial for monitoring the disease. However, CSF GFAP does not have substantial discriminating potential, and further wide-scale studies are necessary to confirm these results. The diagnostic utility of CSF neurofilament for Alzheimer’s disease is limited due to its low specificity, and there is no association with CSF Aβ_42_, suggesting that it might show neurodegeneration independently of Aβ pathology.

Studies have provided evidence that CSF α-synuclein and TDP-43 proteinopathy may be potentially useful in the diagnosis and development of Alzheimer’s disease. These findings increase the value of CSF TDP-43 as a biomarker for diagnosis and prognosis of Alzheimer’s disease; research in this area is ongoing. The relationship between CSF α-synuclein and established core biomarkers such as Aβ_42_ and p-tau could advance its diagnostic accuracy and significantly discriminate Alzheimer’s disease with primary α-synucleinopathies.

The data on CSF BACE1 protein and its activity are promising and show that these two tests are elevated in Alzheimer’s disease patients compared to elderly health controls. CSF BACE1 activity and its protein levels could be utilized for the early diagnosis and progression of Alzheimer’s disease. However, substantial research comprising large populations is needed to confirm these findings.

Using the real-time PCR method enables numerous miRNAs to be recognized and their altered expressions to be detected at different stages of Alzheimer’s disease; these miRNAs may be involved in the pathogenesis of the condition. Total CSF and CSF-derived exosome miRNAs can be combined, which improves diagnostic sensitivity and specificity. More effective molecular methods that are ultrasensitive may be employed to increase detection. Validation of the large number of miRNAs identified in large prospective cohort studies of Alzheimer’s disease patients is warranted.

The core biomarkers for Alzheimer’s disease are most commonly determined by ELISA, the gold-standard method, and there are data that demonstrate its validation and satisfactory diagnostic performance. However, there is variability in measurements of these CSF core biomarkers among laboratories due to different analytical procedures, variations in calibrator and reagent quality from manufacturers, and biological inconsistencies that influence the biomarker concentration. These issues can be resolved by standardization efforts involving the implementation of certified reference materials and the use of ELISA platforms that are fully automated. With the increasing use of mass spectrometry to measure core CSF biomarkers, these procedures should be fully validated, and there should be the application of certified reference standards in order to maximize their sensitivity and reliability.

The novel biomarkers described have all been utilized in research trials, and moving them to clinical diagnostic testing will require well-designed large prospective clinical studies to assess their efficacy and compare them with current gold-standards. Clinical-grade assays for novel CSF biomarkers should be developed on platforms that are validated and fully automated so that the measurements are reliable, reproducible, and accurate. This will enable the determination of reference intervals of cut-off points and the general utilization of these novel CSF biomarkers in routine settings for the clinical assessment of individuals with suspected Alzheimer’s disease.

## Figures and Tables

**Table 1:** Pathological mechanisms (Aβ and tau pathology) and associated biomarkers of Alzheimer’s disease and the findings of related studies.

Pathological Mechanism	Biomarker	Study Design Cohort—Participants Main Findings	Main Findings	References
Aβ pathology	Aβ_42_	Meta-analysis: 231 studies, including 13,018 controls and 15,699 patients with Alzheimer’s disease	Aβ_42_ concentration discriminated between those with the disease and controls (Average ratio: 0.56, 95% CI: 0.55–0.58, *p* < 0.0001) Aβ_42_ concentration discriminated between MCI due to AD from stable MCI (Average ratio: 0.67)	[27]
Tau pathology	t-tau	Meta-analysis: 13,018 normal controls 15,699 Alzheimer’s disease patients	CSF t-tau had good diagnostic performance in differentiating both groups (average ratio: 2.54, 95% CI: 2.44–2.64, *p* < 0.0001)	[27]
Tau pathology	p-tau	Meta-analysis: 13,018 normal controls 15,699 Alzheimer’s disease patients	CSF t-tau had good diagnostic performance in differentiating both groups (average ratio 1.88, 95% CI:1.79–1.97, *p* < 0.0001)	[27]
Aβ pathology	Aβ_42_	Meta-analysis	Low CSF Aβ_42_ levels (SMD: −1.57, CI 95%: −2.30 to −0.84, *p* < 0.001) predict conversion from MCI to AD	[28]
Aβ pathology	Aβ_42_/Aβ_40_ ratio	Observational: 69 patients with AD, 26 patients with VaD, 16 patients with DLB, 27 patients with FTD, and 47 controls.	CSF Aβ_42_/Aβ_40_ ratio improves differentiation of AD patients from VaD, DLB, and non-AD dementia patients when compared to only Aβ_42_	[29]
Aβ pathology	Aβ_42_/Aβ_40_ ratio	Observational: 17 FTD, 17 DLB and 16 with vascular dementia.	Aβ_40_/Aβ_42_ ratio improved diagnostic presentation of Aβ_42_ in differentiating AD from FTD, DLB, and VaD	[30]
Aβ pathology	BACE1 activity	Case–control and longitudinal follow-up	Subjects with MCI who progressed to AD had higher baseline BACE1 activity than those with stable MCI and subjects with MCI who developed other forms of dementia	[31]
Aβ pathology	BACE1 activity	Observational: 30 AD patients and 19 healthy controls	BACE1 activity and protein levels were significantly increased in AD compared to 19 elderly healthy controls.	[32]
Tau pathology	t-tau	Observational: 30 patients with MCI followed for two years	Sensitivity of 82% (95% CI: 76–86%) and a specificity of 70% (95% CI: 65–75%) in differentiating AD from MCI	[33]
Tau pathology	t-tau	Meta-analysis	Lower tau concentrations differentiated DLB (sensitivity 73%, specificity 90%, FTLD (sensitivity and specificity 74%), VaD (sensitivity 73%, specificity 86%)	[34]
Tau pathology	t-tau and Aβ_42_	150 AD, 100 healthy volunteers, 84 patients with other neurologic disorders, 79 no-Alzheimer’s types of dementia	Aβ_42_ and tau (85% sensitivity, specificity 86%; 95% CI: 81–91%) compared with Aβ_42_ (specificity 55%; 95% CI: 47–62%) and t-tau (specificity 65%; 95% CI: 58–72%) in differentiating AD from others	[35]
Tau pathology	p-tau/Aβ_42_ and t-tau/Aβ_40_ ratios	Longitudinal follow-up (9–13 years) and postmortem evaluation of 227 subjects with AD (97% autopsy-confirmed), MCI, other dementia syndromes, and controls	p-tau/Aβ_42_ (sensitivity of 94% and specificity of 90%) and t-tau/Aβ_40_ ratios (sensitivity of 92% and specificity of 94%) highly discriminate autopsy-confirmed Alzheimer’s disease from controls	[36]

## Data Availability

Not applicable.
